# Supplementary material for: General practitioner and nurse practitioner attitudes towards electronic reminders in primary care: a qualitative analysis
Source: BMJ Open. 2021 Jul 12;11(7):e045050. doi: 10.1136/bmjopen-2020-045050 (PMC8276294; doi:10.1136/bmjopen-2020-045050)
Supplement: Supplementary data [file bmjopen-2020-045050supp001.pdf]

Version no: 1 19.01.18

Imperial College London

Participant information sheet

Title of Project: Evaluating reminders in primary care electronic patient records

IRAS No: 234951

You are being invited to take part in a research study to evaluate GPs and practice nurses' attitudes towards reminders in electronic patient records. Before you decide it is important for you to understand why the research is being done and what it will involve. Please take time to read the following information carefully and discuss it with others if you wish.
Ask us if there is anything that is not clear or if you would like more information. Take time to decide whether or not you wish to take part.
Thank you for reading this.

Who will conduct the research?

This research is being conducted by Dr Elizabeth Cecil (Senior Researcher) and Professor Paul Aylin (Chief Investigator). The study is part of the Imperial Patient Safety Translational Research Centre's scheme of research. Development of the research has been supported and guided by a group clinicians, patients and researchers with an interest in primary care patient safety.

What is the purpose of the study?

Primary care administrative systems offer opportunity to improve patient care in the form of delivering reminders to primary health care professionals. An electronic reminder is a pop-up prompt created within a clinical administrative system at the point of care. These reminders remind a primary health care professional of an action required to provide good quality care or prevent harm to the patient. Examples of reminders are those that recommend: safe prescribing of medications (and warning of possible interactions); to provide advice on a preventive or clinical intervention (such as smoking cessation or immunisation); or to document clinical information (such as blood pressure). Attitudes to and the impact of reminders have been studied in the US, but little is known about attitudes in the UK. This research will inform further projects within Patient Safety and Translation Research Centre's programme of work to improve administrative systems, which will ultimately contribute to a safer NHS.

Why have I been chosen?

You have been chosen because as a primary health care professional you have extensive experience of using a primary care administrative system and therefore will have important insights into their electronic reminders. For this study we expect to recruit up to 25 further primary care nurses and GPs including partners and salaried GPs who will help us to get a rounded picture of the attitudes towards electronic reminders in primary care.

Do I have to take part?

No. It is up to you to decide whether or not to take part. If you do decide to take part you will be given this information sheet to keep and be asked to sign a consent form. If you decide to take part you are still free to withdraw at any time and without giving a reason.

What will happen to me if I take part?

If you decide to take part you will be asked a series of open ended questions to assess your views on reminders, which will be recorded for analysis. These questions will asked in a single interview, which can take place in a setting of your choice and will take a maximum of 1hr of your time (including the brief, consent and debrief). You will be reimbursed for your time and travel.

Version no: 1 19.01.18

What if something goes wrong?

If you are harmed by taking part in this research project, there are no special compensation arrangements. If you are harmed due to someone's negligence, then you may have grounds for a legal action. Regardless of this, if you wish to complain, or have any concerns about any aspect of the way you have been treated during the course of this study then you should immediately inform the senior researcher (e.cecil@imperial.ac.uk). The normal National Health Service complaints mechanisms are also available to you. If you are still not satisfied with the response, you may contact the Imperial AHSC Joint Research Compliance Office.

What are we studying

We are investigating attitudes towards reminders in primary care administrative systems. A reminder is a pop-up prompt created within a clinical administrative system at the point of care. These reminders remind a primary health care professional of an action required to provide good quality care or prevent harm to the patient. Examples of reminders are those that recommend: safe prescribing of medications (and warning of possible interactions); to provide advice on a preventive or clinical intervention (such as smoking cessation or immunisation); or to document clinical information (such as blood pressure).

What are the possible disadvantages of taking part?

We know that as a practising GP/Nurse your time is a limited. We will do our utmost not to inconvenience you and will ask for only 45 minutes of your time.

What are the possible benefits of taking part?

We cannot promise the study will directly affect your relationship with reminders in primary care, but the information could help to improve clinical systems which will ultimately contribute to a safer NHS.

Will my taking part in this study be kept confidential?

All information collected in both the consent form and the interview data will be kept strictly confidential. You will be assigned a code and only the senior researcher will have the key to identify participants. Personal data contained within the consent form will only be available to direct members of the study team and will be stored within the restricted secured networked IT system at Imperial College for 10 years after the completion of the study. The audio recordings of the interviews will be transcribed by a transcription company, Page Six, who have a confidentiality agreement in place with Imperial College London. The original recordings will be destroyed after transcription. Our procedures for handling, processing, storage and destruction of their data are compliant with the Data Protection Act 1998.

What will happen to the results of the research study?

Interviews will be anonymised using thematic analysis. Results will be written up into reports and research articles. These will include anonymised quotes from interviews. You will be notified of these before publication.

Who is organising and funding the research?

This study is part of the work of the Patient Safety and Translational Research Centre at Imperial College London (PSTRC). PSTRC is funded by the National Institute for Health Research.

Who has reviewed the study?

This study was reviewed by members of the PSTRC Research Partnership Group which comprises of patients and members of the general public, on 19.12.17. The Study Coordination Centre has obtained approval from the Health Regulator Authority (HRA).

Who should I contact for further information?

The senior researcher, Elizabeth Cecil can be contacted at:

e.cecil@imperial.ac.uk
020 7594 1838

Imperial College London,
Department of Primary Care and Public Health, Reynolds Building, St Dunstan's Road
London W6 8RP

Version no: 1 19.01.18

Imperial College London

Topic Guide

Title of Project: Evaluating reminders in primary care electronic patient records

IRAS No: 234951

Design: Semi-structured interview

Objectives:

- To assess GPs and practice nurses' familiarity with reminders in electronic health records.
- To determine GPs and practice nurses' views on the relevance of information provided by reminders in electronic health records.
- To understand the factors involved in why GPs and practice nurses ignore some reminders and not others.
- To explore how GPs and practice nurses feel reminders can be improved.

Brief:

Thank you very much for taking part in this study. My name is Elizabeth Cecil I work at Imperial College London. As you know we are carrying out a project to assess the primary health care professionals' views on reminders in electronic health records in primary care.

An electronic reminder is a pop-up prompt created within a clinical administrative system at the point of care. These reminders remind a primary health care professional of an action required to provide good quality care or prevent harm to the patient. Examples of reminders are those that recommend: safe prescribing of medications (and warning of possible interactions); to provide advice on a preventive or clinical intervention (such as smoking cessation or immunisation); or to document clinical information (such as blood pressure).

The interview is very informal and confidential. Only me and the direct members of my team will see or hear it. Your name will not appear on anything that we write and you will be notified before we publish the results. With your permission I would like to record the interview. This so that I can concentrate on your responses rather be distracted by taking notes. All recordings will be destroyed once the project is complete. Right, we'll both sign the two consent forms and there is a copy for you and one for me. Thank you we'll start. *Start taping.*

Version no: 1 19.01.18

Questions	Possible probes
**I would like to start by collecting some information on you and the practice**	
Please confirm your participant ID	It's written on your consent form
What is your position at this practice?	Are you a salaried GP, GP partner or practice nurse?
How long have you been qualified?	How long as a GP/practice nurse
What EHR system is used at this practice?	
Do you feel confident using the EHR system in your clinical practice?	Compared to your colleagues how competent do you feel you are?
**I now wish to discuss your familiarity with electronic reminders (knowledge)**	
For this research we define a reminder (in electronic primary care patient records) as: **A prompt created within a clinical administrative system at the point of care which reminds a primary health care professional of an required or desired action.**	
Can you give me some examples of reminders that you come across in your working day?	Are these the most common reminders
How many reminders do you tend to come across in a single day?	What factors are related to the number of reminders? E.g. time of year, patient age, comorbidities
Do reminders come up at other times than at the point of care, for example when the GP or administrator are adding patient information into the EHR?	Who is ultimately responsible for the outcome of a reminder if it does not always appear at point of care?
**(Skills)**<br>Do you feel confident to effectively manage reminders?<br><br>Have you been given training on how to respond to reminders?	Is the training the GPs receive in the surgery or in their training elsewhere?
**Now I wish to explore your attitudes to reminders (beliefs – self-standards)**	
What do you think about reminders?	
How credible or relevant do you find the information provided by reminders systems?	
Can you give me an example of a reminder that you feel is particularly useful/relevant?	Why are they useful?<br>How much do you rely on these prompts?
**(To explore behaviour towards reminders / intentions)**	(Emotional response)

Version no: 1 19.01.18

What is your immediate reaction to a reminder when it appears on your screen?	How does this make you feel e.g (delight, stress, dread)
Are there some reminders you are more likely to override/ignore than other others?	Why do you override/ignore this reminder compared with other reminders?
Can you expand on what factors dictate you to override/ignore?	For example are you more likely to ignore at the end of a day/week
Are there some reminders you never override/ignore?	Can you expand on why?
How often do you override/ignore reminders?	How many would that be, say, out of 10?
**(Beliefs about consequences)**<br>What will happen if you ignore a reminder?	
Can you give any example of a negative outcome after ignoring an alert?<br>How was this handled and was anything learned after the event?	If not by you but by any of your colleagues?
**(Social influences)**<br>To what extent does practice management or your peers influence your response to reminders?	
**(Decision processes)**<br>Do you feel that reminders benefit the care you provide in the consultation?	Do the reminders help you with the decisions you make on treating the patient?
Do you think there is convincing evidence to support the use of reminders in electronic health records?	

You have given me a detailed description of your attitudes towards reminders.

Do you think your colleagues both within your practice and within the profession feel the same way?	What dictates why they feel differently about reminders?
**(Constraints)**<br>What additional resources do you feel could help you effectively respond to a reminder?	Time is an obvious constraint but are there others?

Lets discuss your opinions on how to improve reminders

How would you improve the reminders if you could?	What specific changes do you feel would help facilitate the use of electronic reminders?
Do you feel that a redesign should include a ranking system?	For example colour coding for importance.
What information do you feel needs to be included that may have previously been missed?	

Version no: 1 19.01.18

Do you think these improvements will change your behaviour to and attitudes towards reminders?	Will you be less likely to ignore a reminder?

The Patient Safety and Translational Research Centre is carrying out research identify determinants of deterioration in primary care patients using EHRs.

Do you think that there are already reminders that can help to identify deteriorating patients or those patients where a diagnoses has previously been missed?	If not a reminder what other tools do you use?
Do you think that a reminder to flag up a deteriorating patient would be important?	

Debrief

Thank you for taking part. Do you have any questions that you want to ask me? If you do need to get hold of me in the future, my contact details are on the information sheet.
