# Supplementary material for: General practitioner and nurse practitioner attitudes towards electronic reminders in primary care: a qualitative analysis
Source: BMJ Open. 2021 Jul 12;11(7):e045050. doi: 10.1136/bmjopen-2020-045050 (PMC8276294; doi:10.1136/bmjopen-2020-045050)

Supplementary Figure 1: Thematic map of general practitioner and nurse practitioner views of electronic reminders

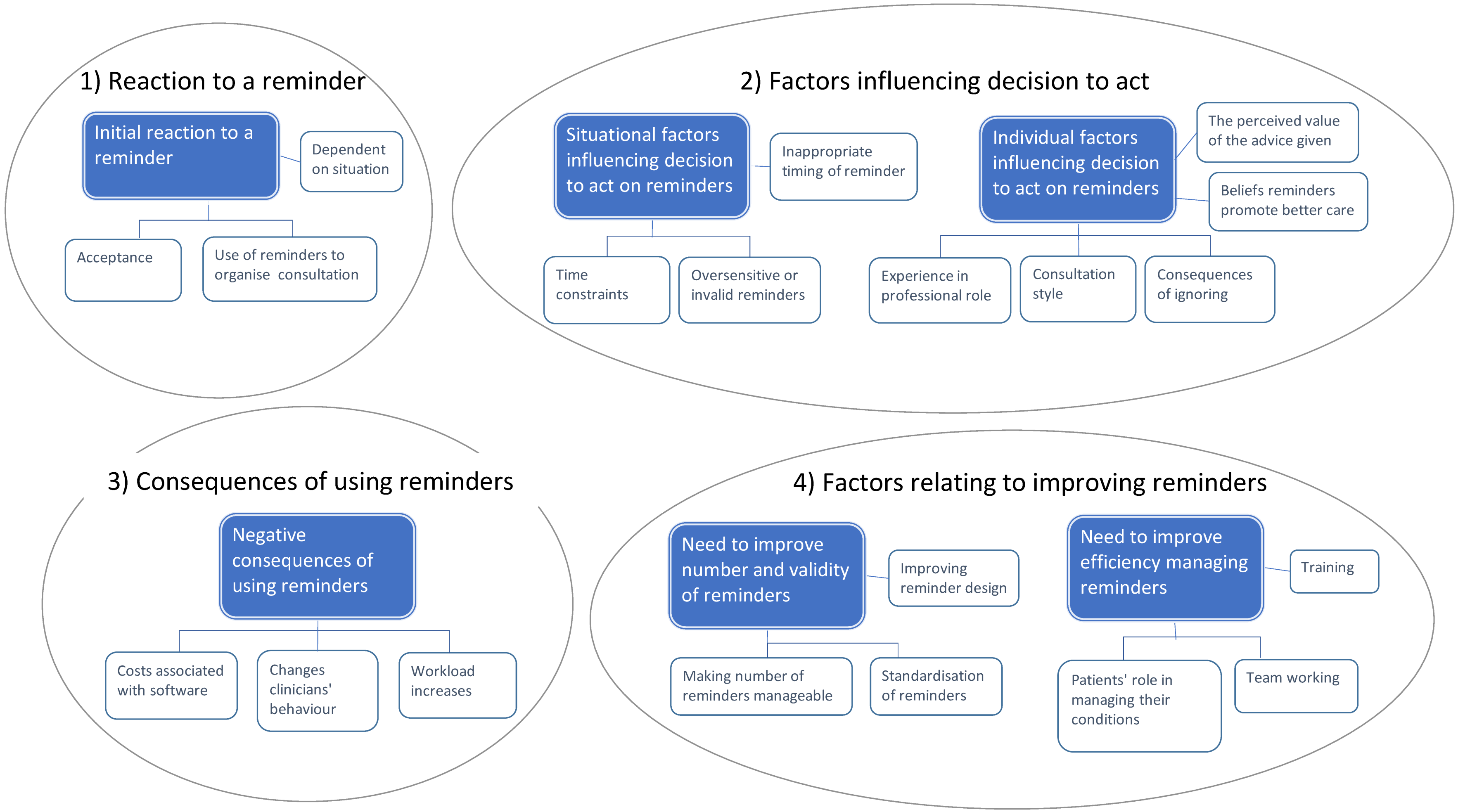

Supplement: Supplementary data [file bmjopen-2020-045050supp003.pdf]
