# Supplementary material for: General practitioner and nurse practitioner attitudes towards electronic reminders in primary care: a qualitative analysis
Source: BMJ Open. 2021 Jul 12;11(7):e045050. doi: 10.1136/bmjopen-2020-045050 (PMC8276294; doi:10.1136/bmjopen-2020-045050)
Supplement: Supplementary data [file bmjopen-2020-045050supp002.pdf]

Themes and subthemes	Examples	Participant
**Reaction to a reminder**		
Acceptance	*I think that at this stage it doesn't make me feel anything because it's there all the time. I think it's become fairly automatic that you glance at it and it depends which one of the many warnings it is.*	[GP Partner, Male, Yorkshire & Humber, EMIS]
Dependent on situation	*If it's a busy clinic, stress, because it's, like, 'Ugh.' If it looks like it's going to be a complicated patient and it's an out-of-hours reminder, then relief probably.*	[GP Partner, Male, Yorkshire & Humber, SystmOne]
Use of reminders to organise consultation	*What I tend to do, and I think most doctors do this, is we kind of do a pre-consultation before the list. So then, I would go through my list, see what reminders come up, and think, okay, fine, supposing it's a child, I'll remember to talk to Mum about reminding about immunisations.*	[GP Partner, Female, NW London, EMIS]
**Individual factors influencing decision to act on reminders**		
The perceived value of the advice given		
Up to date advice	*So, the guidance is very accurate and up-to-date and regularly, it's responsive with regards to the changes that are happening in the BNF to be updated*	[GP Partner, Male, NW London, EMIS]
With infrequent preventive care	*the reminders for cervical smears, because it's three years so some people just don't receive the letters; so promoting them …*	[GP Registrar, Male, NW London, EMIS]
Supportive	*And I think doctors are more confident to ignore the prescribing advice than I would be at all*	
The value of reminders to organise work		*I wouldn't … do half the things that I do if it wasn't for the reminders.*
Beliefs that reminders promote better care		
For continuity of care	*I appreciate them, I think that they have definitely a use because I can't remember everything*	[GP Partner, Female, NW London, EMIS]
For possible diagnoses	*if there's a kind of brain fatigue of seeing so many patients, it's easy sometimes to record, but not necessarily process, what you are writing. That's in any field. So, you're writing, yes, heart rate 150, I know that's high but I haven't really thought about it. So, if the sepsis alert comes up, I think it's good because it also has made you think.*	[GP Partner, Male, NW London, EMIS]
Experience in professional role, eg. Clinical interests or years in clinician practice		
Beliefs of one's personal capabilities	*I mean, I'd be worried if a GP at this stage wasn't aware of sepsis*	[Salaried GP, Female, NW London, SystmOne]

Beliefs of others confidence capabilities		[Nurse Practitioner, Female, NW London, SystmOne]
Feelings of being in control	*I will try, like this morning, I've got quite a few I've done the whole lot of, and I feel really satisfied.*	[Nurse Practitioner, Female, NW London]
Interests and belief in importance of reminder	*everyone's the same, their interests or their increased fields of knowledge are the ones that they're sort of going to look at more, so even if they are improved, things like the patients with rheumatoid arthritis and things that need reviews*	[Nurse Practitioner, Female, NW London, EMIS]
Position and importance of reminder	*I think the partners will really try and crack through the things more. One could argue it's because money is involved*	[GP Partner, Female, NW London, EMIS]
Feelings of responsibility	*Doctors …they might ignore the foot check whereas myself or my colleagues, peers, would either do it or tell somebody to go and book an appointment*	[Nurse Practitioner, Female, NW London, SystmOne]
Feelings of guilt	*I will feel guilty, if I haven't asked somebody whether they smoke I feel a twinge of guilt, but clinically it's probably not going to do a great deal*	[Nurse Practitioner, Female, Yorkshire & Humber]
Consultation style		
	Writing up oh I should have done that doesn't matter then whether I'm on time or 20 minutes late because they've still gone out the door.	[GP Partner, Male, Yorkshire & Humber, SystmOne]
Consequences of ignoring reminders		
Litigation	*.... people are very reluctant to make a change because... nobody wants to take responsibility. Everybody's scared of being sued and the computer companies are very scared of being sued... so they overdo it. They absolutely overdo it. And unfortunately that therefore makes the warnings much weaker because they're not specific enough.*	[GP Partner, Male, Yorkshire & Humber, SystmOne]
Litigation	*I'm very risk-averse, and I'm always aware of litigation.*	[Nurse Practitioner & Admin Role, Female, Yorkshire & Humber, SystmOne]
Patient Harm	*if it's about patient safety - and a lot of them are about patient safety - but if it feels a true risk and you ignored it then the consequence is that you would do the patient harm.*	[GP Partner, Male, Yorkshire & Humber]
	… and the patient's not in front of you so to change a medication when they're not there… albeit it's the same drug, it's clinically not… I don't think it's safe. So, yes, I override them.	[GP Registrar, Female, NW London, EMIS
Financial loss	*A lot of them are about money, so ignoring the CCG's instruction to use a cheaper drug, sometimes you might get a rebuke if it's a really, really expensive decision. But that would be as rare as hen's teeth that that would happen.*	[GP Partner, Male, Yorkshire & Humber, EMIS]
Lost opportunity	*example cervical screening, and I can't remember a case off the top of my head. But if you*	[GP Partner, Male, NW London, EMIS]

	ignored it and then you didn't necessarily remind the patient and you think, this is a young person who doesn't really come into the surgery, this was an opportunistic consultation, it would have been a great chance to remind them of that,	
Increase workload	*Well ultimately, you have to call the patient back in to get the work done, so ultimately yes.*	[Nurse Practitioner, Female, NW London, SystmOne]
Audit	*I mean I was told that they do audit who's doing the flus so who's choosing to say, no, come back but then they can look at that and see was it appropriate because obviously if they're unwell*	[Nurse Practitioner, Female, NW London]
Genuine reasons for ignoring reminders	*I would defend – if I got picked up on it, and I wouldn't often – I would be able to defend why I ignored it for that patient.*	[Nurse Practitioner, Female, NW London, SystmOne]
**Situational factors influencing decision to act on reminders**		
Time constraints	*I wouldn't be able to do anything if I didn't ignore the majority of them.*	[GP Partner, Male, Yorkshire & Humber, EMIS]
	That's pretty standard, I mean here they do 10 minutes and every hour you get a 10 minute block so they say that's 12 minutes.	[Nurse Practitioner, Female, NW London, EMIS]
Inappropriateness of reminder's appearance		
Within consultation flow	*I think they're fantastic if they're at the right time.*	[GP Partner, Male, Yorkshire & Humber, SystmOne]
Within consultation flow	*And instead of them just being, for example, a box somewhere it would be nice if the reminder came up at the appropriate time, so if you are doing a history if the appropriate reminder came up in… there was a history based reminder, if the medication one came up in the medication, when you press examination maybe all the examination ones so that height, weight, blood pressure came up at that time so that, you know, you could do it in a more natural flowing*	[GP Partner, Female, NW London, EMIS]
Within consultation flow	*Some of the other prompts tend to come afterwards rather than at the point of care,*	[GP Partner, Male, Yorkshire & Humber, SystmOne]
In relation to reason for patient visit	*It's embarrassing, you've got a patient who's coming in who looks like they're in pain or stress and the first thing you have to do is ask about smoking -*	[GP Partner, Female, NW London, EMIS]
In relation to the time of year	*I'm not going to respond to anything to do with the flu jab at this point in time because it's the wrong time of year.*	[GP Partner, Female, NW London, SystmOne]
	with QOF related indicators there is a timeframe and, … there may be a reminder to do something that was only done two or three months ago, for which there is no real clinical need to do now but because the system is set up in that way..	[GP Partner, Male, NW London, EMIS]

In timing since previous reminder	*And so do you think that a reminder should have a sort of additional algorithm that says basically six months afterwards*	[GP Partner, Male, NW London, EMIS]
Oversensitive or invalid reminders	*Extremely sensitive. So, if you put in something like hay fever, it will come up with a sepsis alert, because it will just catch that bit that you say fever, so a lot of time you end up just going exit, exit, exit and it actually creates more work. I don't think my consultations have ever benefitted from a sepsis alert, never.*	[GP Registrar, Female, NW London, EMIS]
System changes	*So, for example, atrial fibrillation has a new template that's come out on the EMIS and I just had a patient today with atrial fibrillation and I noticed it wasn't working properly. So then, I would say to – I rang the manager to say, "Can we just go through this at one point?" Because I think if I'm having problems with it, we probably are as a group because it doesn't seem to be uploaded properly.*	[GP Partner, Female, NW London, EMIS]
**Negative consequences of using reminders**		
Costs associated with software outway any benefits	*The original people that did the alerts were appalling and there was evidence that the cost of the alerts was way in excess of the savings …. So there's lots of examples in the NHS where we make really poor financial decisions. Popups can be one of those.*	[GP Partner, Male, Yorkshire & Humber, EMIS]
Permanency of data recorded	*And I know other patients, even a doctor colleague who had something put on her child's records because she was widowed and so the child was considered more vulnerable. And fair enough, so somebody else looking out for them and thinking about their needs, but it was put on in such a way that it looked like the child was in need on a protection plan, you know, being mishandled. And she couldn't get it taken off for love nor money even though she was a perfectly competent parent and there was a perfectly reasonable need in that child being more vulnerable because her child's father had just died. So there are pop-ups that can be damaging, actually.*	[GP Partner, Female, NW London, EMIS]
Changes PHCPs behaviour		
Changes in the way data is recorded	*they do change the way you do things. So I gave you the example of the sepsis reminders. It makes you wary of putting certain information in because you know it's going to prompt a reminder.*	[GP Partner, Male, Yorkshire & Humber, SystmOne]
Distracts PHCP from patient centredness	*they've just got to be careful that they're not too much of a burden and too intrusive all the time, because it then does detract you away from your primary objective, which is, kind of, interacting with that patient,*	[GP Partner, Male, Yorkshire & Humber, SystmOne]
Patients aware reminders impact on behaviour		
Damages patient-PHCP relationship	*But a lot of patients also have an awareness that it earns us money. And they will quote that back at you. So, you have to be careful and you have to, you know… I think…*	[Nurse Practitioner & Admin Role, Female, Yorkshire & Humber, SystmOne]

Workload increases as patients consult more	*and it's exactly the same medication just a different brand, so then you're spending more time explaining to them, "It's exactly the same thing". So, as I said, I appreciate why it's there; sometimes I do switch from the generic brand to the branded because it's whatever is in fashion and it's common, but there are*	[GP Partner, Male, NW London, EMIS]
Learning from mistakes	*It's not about blame. How can we avoid this? And we put strategies*	[Nurse Practitioner, Female, Yorkshire & Humber, SystmOne]
**Need to improve number and validity of reminders**		
Making number of reminders more manageable		
Joining up IT systems across health and social care	*where we're missing information is where things are done outside the practice so, you know, if we had information that was more freely available within the consultation that would prevent duplication, errors, all sorts of things*	[GP Partner, Female, NW London, EMIS]
Intelligent algorithms	*It's a bit annoying because something like that a lot of the alerts will come up if there's ever been a problem so you look back and you think oh I might actually ask the patient saying about your, you know, liver function and they'll go, 'Oh that was four years ago and it's all fine now and I used to drink and I don't anymore' but EMIS… I don't know how far back EMIS goes but it seems to pick up anything abnormal but you have to check it.*	[Nurse Practitioner, Female, NW London, EMIS]
Patient self-management of condition	*You would hope a lot of them, like blood tests when you're on medication that need to check would like to think that the patient would take the onus themselves and know I've got to do this every three months or I've got to do this every six months but sadly a lot of them…*	[Nurse Practitioner, Female, NW London, EMIS]
Patient self-management of data	*I mean, I think some of the QOF ones, the minor things like smoking and everything, if we go to sort of patient-held IT records and things like this, they will actively put that stuff in for us*	[Nurse Practitioner, Female, Yorkshire & Humber, SystmOne]
Make all reminders valid	*I don't think anybody's ever complained about the serious alerts, it's the infuriating pop ups.*	[GP Partner, Female, NW London, EMIS]
	So, I think if you say proceed, then it doesn't ask anything, but if you say ignore, it comes up saying, basically, why are you ignoring me? So, that can be quite frustrating, especially if you're thinking, you should realise why I'm ignoring this after a while	[GP Partner, Female, NW London, EMIS]
Make reminders more timely	*with QOF related indicators there is a timeframe and, as I said, there may be a reminder to do something that was only done two or three months ago, for which there is no real clinical need to do now but because the system is set up in that way..*	[GP Partner, Male, NW London, EMIS]
Standardisation of reminders	*I'd like an NHS formulary that was set nationally*	[GP Partner, Female, NW London, EMIS]

**Need to improve the validity of reminders**		
Improving the evidence base of reminders	*QOF, the evidence-base for that is poor, you know, not robust. And I expect that the evidence-base for this will be mixed. But I don't think it's going to mean that they go away.*	[Nurse Practitioner, Female, Yorkshire & Humber, SystmOne]
Reminders force incorrect data entry	*So, because the* CCG [Clinical Commissioning Group] *will be looking at our referral letters we should get patient's consent to allow them to look at it, that virtually never happens but you have to tick the box*	[GP Partner, Male, Yorkshire & Humber, SystmOne
Need to improve efficiency managing reminders		
Training	*… no, sometimes extra training to know what's the quickest way of doing something, …., so if it's recording, say, a vaccination that you've given, it might say that they need a B12 is it quicker to use templates?*	[GP Partner, Female, NW London, EMIS]
	…. I don't think it was long enough, the training, because actually EMIS is your… well, computer system is how you do everything; it's your note system, it's how you communicate with other healthcare professionals, it's your referral system, and you learn to use it very superficially	[GP Registrar, Female, NW London, EMIS]
**Need to improve the design of reminders**		
Use of drop down	*and then you could actually have something saying it's an ENT, it's a chest, it's an acute abdomen, it's a urine, you know, and then you could just quickly pick which one you want and then you could put that information in because the advantages of that is that those would be just a tick box exercise whereas when you add it yourself you would put in urine analysis then you have a drop down then you have to find it, you know? So, you could just free text it but that's ridiculous, you don't want to free text anything, you want to read code as much as you possibly can.*	[GP Partner, Female, NW London, EMIS]
Better use of icons	*They could be personalised, not quite as funkily as this where it's very obvious to me within a flash that this patient smokes - she uses an inhaler and she's probably got restrictive or obstructive or interstitial lung disease - that she's got brittle bones, well she's definitely got lung disease, that she's at risk for dementia because (overspeaking), that she might not have had her flu jab. I mean, I can tell very, very quickly. I never need to ask somebody whether they smoke or not; I just look at the screen...So the icons are quite useful in that respect.*	[GP Partner, Female, NW London, SystmOne]
Need to change attitudes	*I think the alerts are just going to be annoying going forward until we as clinicians change the way that we have to input data*	[GP Registrar, Female, NW London, EMIS]
Ranking in reminders isn't very easy	*Ranking on what basis? It's hard to know whether a patient's allergy is more important than a child's protection need or a patient's, you know, medication review*	[GP Partner, Female, NW London, SystmOne]

Reorganisation of chronic disease care	*At the moment, there's a real kind of shift in what a lot of GPs are doing at the moment, so it's moved from, historically, a lot of chronic disease management and now it's a lot of acute care. It's getting increasingly difficult to be both kinds of doctor in the ten minutes. So, to respond to reminders, in theory, for the best interests of patients, you've got to be thinking, can chronic disease management be managed in a different way with stable patients, it's like almost a chronic disease birthday or something like that where the patient thinks, right, every year in the month of my birthday, the practice is going to deal with all my reminders to do with diabetes or whatever chronic disease it is. It's so difficult to be an acute care clinician plus chronic disease manager at the same time, and that's where alerts sometimes probably get missed or not missed, but consciously avoided. So, an appropriate kind of management of the resource in terms of thinking, right, do we need to have more acute care clinicians or chronic disease managers?*	[GP Partner, Male, NW London, EMIS]
