# Supplementary material for: General practitioner and nurse practitioner attitudes towards electronic reminders in primary care: a qualitative analysis
Source: BMJ Open. 2021 Jul 12;11(7):e045050. doi: 10.1136/bmjopen-2020-045050 (PMC8276294; doi:10.1136/bmjopen-2020-045050)
Supplement: Author's manuscript [file bmjopen-2020-045050.draft_revisions.pdf]

BMJ Open is committed to open peer review. As part of this commitment we make the peer review history of every article we publish publicly available.

When an article is published we post the peer reviewers' comments and the authors' responses online. We also post the versions of the paper that were used during peer review. These are the versions that the peer review comments apply to.

The versions of the paper that follow are the versions that were submitted during the peer review process. They are not the versions of record or the final published versions. They should not be cited or distributed as the published version of this manuscript.

BMJ Open is an open access journal and the full, final, typeset and author-corrected version of record of the manuscript is available on our site with no access controls, subscription charges or pay-per-view fees (http://bmjopen.bmj.com).

If you have any questions on BMJ Open's open peer review process please email info.bmjopen@bmj.com

BMJ Open

General practitioner and practice nurse attitudes towards electronic reminders in primary care: A qualitative analysis

Journal:	*BMJ Open*
Manuscript ID	bmjopen-2020-045050
Article Type:	Original research
Date Submitted by the Author:	22-Sep-2020

Complete List of Authors:	Cecil, E; Imperial College London, Primary Care and Public Health<br>Dewa, Lindsay; Imperial College London, School of Public Health; Imperial College London, Patient Safety Translational Research Centre<br>Ma, Richard; Imperial College London, Primary Care and Public Health<br>Majeed, Azeem; Imperial College London, Primary Care<br>Aylin, Paul; Imperial College London, Primary Care and Public Health

Keywords:	PRIMARY CARE, Information technology < BIOTECHNOLOGY & BIOINFORMATICS, Quality in health care < HEALTH SERVICES ADMINISTRATION & MANAGEMENT

SCHOLARONE™
Manuscripts

I, the Submitting Author has the right to grant and does grant on behalf of all authors of the Work (as defined in the below author licence), an exclusive licence and/or a non-exclusive licence for contributions from authors who are: i) UK Crown employees; ii) where BMJ has agreed a CC-BY licence shall apply, and/or iii) in accordance with the terms applicable for US Federal Government officers or employees acting as part of their official duties; on a worldwide, perpetual, irrevocable, royalty-free basis to BMJ Publishing Group Ltd ("BMJ") its licensees and where the relevant Journal is co-owned by BMJ to the co-owners of the Journal, to publish the Work in this journal and any other BMJ products and to exploit all rights, as set out in our licence.

The Submitting Author accepts and understands that any supply made under these terms is made by BMJ to the Submitting Author unless you are acting as an employee on behalf of your employer or a postgraduate student of an affiliated institution which is paying any applicable article publishing charge ("APC") for Open Access articles. Where the Submitting Author wishes to make the Work available on an Open Access basis (and intends to pay the relevant APC), the terms of reuse of such Open Access shall be governed by a Creative Commons licence – details of these licences and which Creative Commons licence will apply to this Work are set out in our licence referred to above.

Other than as permitted in any relevant BMJ Author's Self Archiving Policies, I confirm this Work has not been accepted for publication elsewhere, is not being considered for publication elsewhere and does not duplicate material already published. I confirm all authors consent to publication of this Work and authorise the granting of this licence.

For peer review only

General practitioner and practice nurse attitudes towards electronic reminders in primary care: A qualitative analysis

Elizabeth Cecil, Lindsay Dewa, Richard Ma, Azeem Majeed, Paul Aylin

The Dr Foster Unit, Department of Primary Care and Public Health, Imperial College London, 3 Dorset Rise, London EC4Y 8EN, UK
Elizabeth Cecil, Research Associate
Lindsay Dewa, Research Associate
Paul Aylin, Professor of Epidemiology and Public Health

Department of Primary Care and Public Health, Imperial College London, W6 8RP, UK
Richard Ma, NIHR Doctoral Research Fellow (Clinical)
Azeem Majeed, Professor of Primary Care

Correspondence to Elizabeth Cecil: e.cecil@imperial.ac.uk, +44 2075941838

Acknowledgements

We wish to thank Puja Verma, a GP in North West London, for her support developing the topic guide. To members of the Research Partners group for their contribution in the project development and result interpretation. A thank you to Sean Shillito, North West London CRN and Michelle Platton, Yorkshire and Humber CRN for their support in recruiting participants for this study.
Funding
This study was funded by National Institute for Health Research (NIHR) Patient Safety Translational Research Centre (PSTRC) programme grant (PSTRC-2016-004). RM is funded by a NIHR Doctoral Research Fellowship (DRF-2017-10-181) for this research project. The Dr Foster Unit is an academic unit in the Department of Primary Care and Public Health, within the School of Public Health, Imperial College London. The unit receives research funding from the National Institute for Health Research and Dr Foster Intelligence, an independent health service research organisation (a wholly owned subsidiary of Telstra). The Dr Foster Unit at Imperial is affiliated with the National Institute of Health Research (NIHR) Imperial Patient Safety Translational Research Centre. The NIHR Imperial Patient Safety Translational Centre is a partnership between the Imperial College Healthcare NHS Trust and Imperial College London. Imperial College London is grateful for support from the NW London NIHR Collaboration for Leadership in Applied Health Research & Care (CLAHRC) and the Imperial NIHR Biomedical Research Centre. This paper presents independent research. The views expressed in this publication are those of the authors and not necessarily those of the NHS, the NIHR or the Department of Health and Social Care.

For peer review only - http://bmjopen.bmj.com/site/about/guidelines.xhtml

Conflicts of interest

All authors have completed the Unified Competing Interest form (available on request from the corresponding author), and PA and AB declare that they are partially funded by grants from Dr Foster Intelligence, an independent healthcare information company.

Contributors

All authors contributed to the conception and design of this study. EC, LD and RM carried out the analysis. All authors took part in interpreting the data from this study. All authors commented on and helped to revise drafts of this paper. All authors have approved the final version

For peer review only

For peer review only - http://bmjopen.bmj.com/site/about/guidelines.xhtml

Abstract

Objectives

Reminders in primary care administrative systems aim to help clinicians provide evidence-based care, prescribe safely and save money. However, increased use of reminders can lead to alert fatigue. Our study aimed to assess general practitioners (GP) and practice nurses' (PN) views on electronic reminders in primary care.

Design

A qualitative analysis using semi-structured interviews.

Setting and Participants

Fifteen GPs and PN based in general practices located in North-West London and Yorkshire, England.

Methods

We collected data on participants' views on; i) perceptions of the value of information provided; ii) reminder-related behaviours and iii) how to improve reminders. We carried out a thematic analysis.

Results

Participants were familiar with reminders in their clinical systems and felt many were important to support their clinical work. However, participants reported, on average, 70% of reminders were ignored. Four major themes emerged. 1) Reaction to a reminder, which was mixed and varied by situation. 2) Factors influencing the decision to act on reminders, often related to experience, consultation styles, and interests of participants. Time constraints, alert design, inappropriate presentation, and litigation were also factors. 3) Negative consequences of using reminders were increased workload or costs, and compromising GP and PNs behaviour. 4) Factors relating to improving users' engagement with reminders, were prevention of unnecessary reminders though data linkage across healthcare administrative systems or the development of more intelligent algorithms. Participants felt training was vital to effectively manage reminders.

Conclusions

GPs and PNs believe reminders are useful in supporting the provision of good quality patient care. Improving GPs and PNs' engagement with reminders centres on further developing their relevance to their clinical practice which is personalised, considers cognitive workflow and suppresses inappropriate presentation.

For peer review only

Strengths and limitations of this study

- Our use of in-depth qualitative interviews has produced novel and varied insights into GP and practice nurses' attitudes toward reminders in electronic health records.
- The views and experiences we collected from GP and practice nurses in varying positions based in practices of two regions (North West London, and Yorkshire and Humber) with varying demographic populations has provided a working hypothesis that, we feel, is transferable across GP and practice nurses working within the English NHS.
- We limited researcher bias by using a peer-reviewed topic guide; having two researchers to validate coding and co-develop the thematic map.
- However, we do acknowledge that parts of the knowledge 'map' may be missing.

For peer review only

Introduction

Good quality health care is safe, effective and patient centred.[1] Primary care administrative systems offer the opportunity to improve patient care by delivering reminders to clinicians at the point of care. An electronic reminder is a prompt created within a clinical administrative system, through the interrogation of recorded patient data. They remind the clinician of an action required, which may include a recommendation on safe medication prescribing (warning of possible interactions); a more cost-effective drug; advice on a preventive or clinical intervention (such as smoking cessation or immunisation); or to document clinical information (such as blood pressure) (Table 1).

There is evidence that clinician behaviours and processes of care provision can be improved by point of care computer reminders, but improvements tend to be small and the features of reminder systems that are associated with clinically worthwhile improvements are difficult to determine.[2] However, in primary care, reminders are believed to be particularly important. This is because within the consultation, time pressures, limited information and distracting cues mean that clinicians' thinking (general practitioners (GP) and practice nurses (PN)), needs to be fast and intuitive rather than systematic.[3] Consequently, there has been an increasing use of reminders for a range of clinical tasks (Table 1).

There are barriers to clinical engagement with the reminders. One such barrier is 'alert fatigue',[4] a potential unintended consequence of the increased number of reminders. Alert fatigue happens when the system user becomes desensitised to the digital reminders, and subsequently fails to engage with them. A literature review (predominantly from the USA and within hospital settings) that investigated reminders safety in medication prescribing, found between 49–96% of alerts were overridden.[4] In 2002 a UK cross-sectional study of primary care clinicians assessed attitudes towards drug interaction reminders and found 1 in 4 (of 236 responses) admitted to frequently overriding drug interaction alerts without properly checking them.[5] Other barriers include lack of user friendliness in the administrative systems; time constraints to address the reminder; and clinicians being unable to see the value in the information provided were all barriers to the acceptance of reminders.[6 7-10]

Most research in this area has been outside the UK,[8, 11, 12] where health systems, such as primary care gate-keeping role, can differ.[13] Whilst studies have examined the development of computerised systems to improve quality in primary care,[14, 15] studies tend to be quantitative. An up to date understanding on how GPs and PNs interact with these systems within the UK is lacking. This study aims to address this gap using semi-structured interviews to explore GP and PNs attitudes towards electronic reminders in primary care, to support their enhancement.

For peer review only - http://bmjopen.bmj.com/site/about/guidelines.xhtml

Table 1: Types of reminders in primary care clinical computer systems

Reminder type	Software	Purpose	Viewed	Action required	Modal*
**Drug price comparison alert**	Prescribing decision support software e.g. ScriptSwitch, OptimiseRx	To save on costs of prescribed drugs	A pop-up viewed when prescribing suggesting a cheaper alternative	Action is required to close the pop-up. Information can be ignored	✓
**Drug interaction/monitoring alert**	GP system standard template. Based on UK pharmaceutical reference publications Algorithm draws on patient prescription data	To promote safe prescribing	A pop-up viewed when prescribing warning of potential drug interactions	Action is required to close the pop-up. Information can be ignored	✓
**Allergy alert**	GP system standard template Algorithm draws on patient clinical data	To promote safe prescribing	A pop-up viewed when prescribing warning of potential allergies	Action is required to close the pop-up. Information can be ignored	✓
**Diagnosis alert**	Diagnosis decision support reminder e.g. Sepsis	To increase awareness of condition as a possible diagnosis	A pop-up viewed when entering diagnosis related patient symptoms	Data must be entered to close the pop-up	✓
**Quality care standard reminders (e.g.Quality Outcomes Framework, Flu vaccine)**	GP system templates and toolkits; Algorithms draw on patient clinical data	To promote evidence-based care and maximise financial payment for performance	On the patient home page and viewed when patient record is opened. Information continues to appears as icons when records are updated	Most reminders don't have to be actioned and can be ignored	X
**Additional patient information alerts from set reminder**	Personally created from set template e.g. Safeguarding children template (Royal College of General Practice)	To communicate patient specific information to other clinicians. To create a registry	On the patient home page and viewed when patient record is opened to those granted access	No action required when viewing reminder	X

For peer review only - http://bmjopen.bmj.com/site/about/guidelines.xhtml

For peer review only

**Additional patient information from generic reminder**	Personally created from a generic template or free text e.g. highlighting a patient with additional needs	To communicate patient specific information to oneself or to other clinicians and practice staff	On the patient home page and viewed when patient record is opened to those granted access	No action required when viewing reminder	X

* Modal alerts are those where the system shuts down and becomes unobtainable behind the alert. The user must interacted with the alert in order to return to the patient record.

For peer review only - http://bmjopen.bmj.com/site/about/guidelines.xhtml

Methods

Design

We conducted a qualitative study using semi-structured interviews. The use of semi-structured interviews was chosen because the methodology allows a flexibility to the interview process, which results in the collection of data relating to GP and PNs' attitudes to reminders in electronic health records.

Participants

We recruited a purposeful sample of GPs and PNs from NHS general practices located in two regions in England: North West London (NWL), and Yorkshire and Humber (Y&H) between 12th March and 19th September 2018. These regions were selected to obtain opinions from participants serving populations with varying demographics (e.g. rural compared with urban patients). We selected participants with experience of primary care administrative systems, based on their position, experience and practice population demographics. Only GPs and PNs were approached as they deliver the majority of care within NHS primary care. In UK, GPs can be self-employed (GP Partner) or salaried we approached Partners, Salaried and trainee GPs. PNs have prescribing rights and are likely to face similar challenges, when providing good quality care, as GPs, yet they may have very different attitudes towards the reminders they experience. In the UK, GPs can work Participants were approached through regional National Institute for Health Research (NIHR) Clinical Research Networks (CRN).[16] A researcher [EC] followed up participants who showed an interest and invited them to take part in the study. Recruitment, interviews and data coding took place simultaneously. The number of subjects required for the study was dependent on the data. Recruitment ceased when researchers felt no new major themes were immerging. Sixteen clinicians were approached, one GP (NWL) did not respond, fifteen (94%) took part.

Semi-structured Interviews

A single researcher [EC] conducted the face-to-face semi-structured interviews in a setting of the participant's choice. In most cases (13/15) this was at the participant's practice. The topic guide and participant information sheet were developed through literature;[17, 18] meetings between [EC] and patients with lived experience; external peer review; and was piloted with a single GP based in North West London (Supplementary file 1). The topic guide was structured to explore three main areas: i)

For peer review only - http://bmjopen.bmj.com/site/about/guidelines.xhtml

perceptions of the value of the information provided in the reminder; ii) reminder-related behaviours and iii) how to improve reminders. Participant responses were followed-up with probing questions for additional depth and detail. Interviews lasted between 45-60 minutes and was audio recorded with participant's consent. Participants were paid for their time, which was a set amount determined by CRN guidance. Health Research Authority (HRA) approval was gained (IRAS No: 234951). Ethics approval was not required for this study as participants were NHS staff.

Data analysis

Interviews were anonymised, transcribed verbatim and subjected to a thematic analysis.[19] We went through six stages: 1) familiarisation with data; 2) generating initial codes; 3) searching for themes; 4: reviewing themes; 5) defining and naming themes, and 6) interpretation of themes through consultation with the first, second and third authors (EC, LD, RM). Two researchers (EC and LD) independently coded (stage 2) three transcripts. Eighty percent of codes defined by LD mapped to those defined by EC. Codes that did not map were further examined and recoded if necessary. Codes were then grouped into sub-themes and a thematic map (stage 5) was then developed by researchers [EC, LD, and RM]. We used QSR NVivo 12 to manage data.

Patient and public involvement

We presented the study proposal to a small group of five people, identify as having various health conditions and experience in primary care, as part of an existing advisory group. The patient representatives were reimbursed for their time according to NIHR INVOLVE guidance. The representatives provided feedback on the appropriateness of the research design, research questions and the topic guide. Their comments were incorporated in study development. For example, the patient representatives felt it was important to ascertain competency in using reminders (how confident they felt in using electronic health records and whether they thought they had received adequate training to manage the reminders) as this is likely to be a barrier to reminder engagement. These questions were added to the topic guide. A single patient representative supported interpretation of the study findings.

For peer review only - http://bmjopen.bmj.com/site/about/guidelines.xhtml

Results.

Participants

Fifteen participants were interviewed; eight were female (53%) and two thirds (67%) were GP partners (Table 2). Eight participants used SystmOne (53%) and seven used Egton Medical Information System (EMIS) clinical administrative system (47%). Participants' years of experience within primary care ranged from less than one to 34 years with a median of 18 years. Participants' practices were on average larger than national practices, median population [interquartile range] 9,355 [4,655 to 14,219] vs 6,681 [4,065 to 10,034). They were, on average, slightly more affluent: median Indices of Multiple Deprivation (IMD) scores were 19 [13 to 28] vs 22 [14 to 32] (where higher scores indicate higher deprivation). Twenty percent of practices (2/10) located within rural populations compared with 15% nationally (1248/8248).

Table 2: Characteristics of interview participants and the administrative systems they use

Participant ID	Region	Practice ID	Position	Gender	Experience (years)	Administrative System
2	Y&H	Practice 1	GP Partner	Male	34	EMIS
3	Y&H	Practice 2	GP Partner	Male	18	SystmOne
4	Y&H	Practice 3	GP Partner	Male	13	SystmOne
5	NWL	Practice 4	GP Partner	Female	22	SystmOne
7	Y&H	Practice 5	GP Partner	Male	32	SystmOne
8	Y&H	Practice 6	GP Partner	Male	22	SystmOne
9	Y&H	Practice 6	Nurse Practitioner	Female	30	SystmOne
10	NWL	Practice 7	GP Partner	Female	17	EMIS
11	NWL	Practice 8	GP Partner	Female	13	EMIS
12	NWL	Practice 8	GP Partner	Male	22	EMIS
13	NWL	Practice 9	Nurse Practitioner	Female	28	SystmOne
14	NWL	Practice 10	GP Partner	Male	7	EMIS
15	NWL	Practice 7	Trainee GP	Female	0	EMIS
16	NWL	Practice 4	Salaried GP	Female	2	SystmOne
17	NWL	Practice 7	Nurse Practitioner	Female	15	EMIS

For peer review only

For peer review only - http://bmjopen.bmj.com/site/about/guidelines.xhtml

Study Themes

Participants were familiar with reminders in patients' electronic health records. They reported that reminders were very common and that virtually every patient had at least one occurring during a consultation. The number of reminders varied between patients and were dependent on the patient's health status and time of year.

Our study identified four main themes: 1) reaction to a reminder 2) factors influencing the decision to act on reminders (individual and situational), 3) unintended consequences of using reminders, and 4) factors relating to improving reminders (Figure 1). Theme examples within the interview data are shown in supplementary file 2.

Reaction to a reminder

Participants' initial reaction to a reminder varied. Although participants found reminders frustrating, there was an overall feeling of acceptance towards reminders. Participant's emotional response did, however, depend on the reminder and the situation.

> *If it's a busy clinic, stress, because it's, like, 'Ugh.' If it looks like it's going to be a complicated patient and it's an out-of-hours reminder, then relief probably.*
> [Participant No:9 Nurse Practitioner, Female, Y&H]

Use of reminders to organise the patient consultation

Participants reported reminders had become a routine part of their work; they assessed reminders before a consultation and used the information as a guide to organise and prioritise the patient's needs. They used the reminders to build management plans and to relay information to themselves or colleagues. Participants talked of feelings of satisfaction, accomplishment and being in control after actioning reminders.

For peer review only - http://bmjopen.bmj.com/site/about/guidelines.xhtml

Figure 1: Thematic map of general practitioner and practice nurse views of electronic reminders

For peer review only

For peer review only - http://bmjopen.bmj.com/site/about/guidelines.xhtml

Individual factors influencing the decision to act on reminder

The perceived value of the advice given

Engagement with the reminders varied. Most participants said they would acknowledge the reminder even if they did not act upon it. The majority commonly stated "70%" were ignored.

> *I wouldn't be able to do anything if I didn't ignore the majority of them.*
> [Participant No:2 GP Partner, Male, Y&H]

Generally, participants valued the information provided by the reminders.

> *I don't think anybody's ever complained about the serious alerts, it's the infuriating pop ups*
> [Participant No:10 GP Partner, Female, NWL]

Some of the participants reported that their views on reminders had changed over time, from feeling overwhelmed to acceptance as they became more familiar with them. Most participants valued the advice given by most reminders.

Wth some participants felt that prompting for potentially overlooked diagnoses was particularly helpful in the primary care setting. Due to high patient turnover and short consultation time, some participants believed recording patient information was automatic while processing the information was sometimes more difficult.

Some participants mentioned current reminders were user-friendly and most believed they were evidence-based which meant they could keep up-to-date with guidance; ultimately improving the care they provided. However, a few participants did not share these views and found all reminders intrusive. Overall, the least popular reminders were the drug cost-saving reminders. Participants complained that the price difference between the previously prescribed drug and its alternative was often minimal.

For peer review only - http://bmjopen.bmj.com/site/about/guidelines.xhtml

Experience and professional role

Participants' feelings towards reminders were often dictated by their professional role, clinical interests or years of clinical practice. For example, one reported that he provided out of hours care to patients outside his normal practice and that the most important reminders were those that conveyed additional patient information because they supported continuity of care. A trainee GP also indicated that drug interaction reminders were particularly useful.

> *Everyone's the same, their interests or their increased fields of knowledge are the ones that they're sort of going to look at more*
>
> [Participant No:17 Nurse Practitioner, Female, NWL]

Many participants accepted that colleagues may have different attitudes towards reminders. For example, they suggested that younger clinicians may find managing reminders easier because they tended to be more computer literate. Yet other participants felt older colleagues would find managing reminders easier as they had more experience to process, act on or ignore the information provided.

> *… I think doctors are more confident to ignore the prescribing advice than I would be at all.*
>
> [Participant No:13 Nurse Practitioner, Female, NWL]

There was concern in by more clinically experienced participants that reminders undermined their competence. Others felt that GP partners would be more likely to act on the reminders that had financial rewards. However, some felt that by focusing on these reminders it may make patients more sceptical of the care being provided.

> *I think the partners will really try and crack through the things more. One could argue it's because money is involved.*
>
> [Participant No:10 GP Partner, Female, NWL]

Participants generally felt the responsibility of actioning a reminder lay with the clinician who was with a patient. However, some seemed to feel more emotionally responsible towards managing the reminders. One participant talked about the guilt associated with failing to action a reminder.

For peer review only - http://bmjopen.bmj.com/site/about/guidelines.xhtml

Consultation style

Consultation styles differed across the participants. Some used the computer to support their consultation while others felt that it was important to focus on the patient and only enter data after the patient had left the room. Those who did not use a computer reported that they often missed reminders until it was too late.

> *Writing up oh I should have done that doesn't matter then whether I'm on time or 20 minutes late because they've still gone out the door.*
>
> [Participant No:3 GP Partner, Male, Y&H]

Consequences of ignoring a reminder

Participants generally felt they had genuine reasons for ignoring reminders and would document these decisions.

> *I would defend – if I got picked up on it, and I wouldn't often – I would be able to defend why I ignored it for that patient.*
>
> [Participant No:13 Nurse Practitioner, Female, NWL]

The consequence of ignoring a reminder differed by reminder type. For example, participants reported that ignoring drug cost-saving reminders resulted in (usually small) financial loses, while ignoring drug or allergy reminders could result in a patient safety issue.

> *The only negative outcome is if you ignored an important alert. A lot of them are about money, so ignoring … instruction to use a cheaper drug… you might get a rebuke….Obviously, if it's about patient safety - and a lot of them are about patient safety - but if it feels a true risk and you ignored it then the consequence is that you would do the patient harm.*
>
> [Participant No:2 GP Partner, Male, Y&H]

Most participants reported there were also opportunistic consequences. For example, a consequence was failing to notify young women of the need for a smear test which could be considered a missed opportunity as these patients rarely visit their GP.

Some participants' decision to act was influenced by concerns of the consequences of ignoring a reminder. For instance, their behaviour could be highlighted through audit, or ignoring a reminder

For peer review only - http://bmjopen.bmj.com/site/about/guidelines.xhtml

could lead to a litigation case. PNs felt that they may not get the same support in the case of litigation as GPs and they believed this made them more risk-adverse when managing reminders.

> *I'm very risk-averse, and I'm always aware of litigation …. I'm not convinced that the MDU [Medical Defence Union] would defend me in the same way as they would a GP….*
> [Participant No:9 Nurse Practitioner, Female, Y&H]

No participants had experienced significant events after ignoring a reminder, but a few participants knew others who had.

Situational factors influencing the decision to act on reminders

Number of reminders

Participants felt that there were too many reminders, which led to irritation or stress. Factors identified to increase the number of reminders were patient age, comorbidities or time of year. Information was also repeated. For example, if a patient had more than one comorbid condition then a reminder to take blood pressure may be listed for each condition

> *No, but you end up just clicking cancel 100 times, it just irritates you in the consultation, because you're typing, kind of examining patients, typing, you've got all this like pop up, pop up, pop up, it's just frustrating*
> [Participant No:16 Salaried GP, Female, NWL]

Time constraints

Participants reported they had limited time to manage all reminders in a typical consultation partly due to numbers but also because reminders were time-consuming to action.

Invalid, irrelevant or oversensitive reminders

Participants believed some reminders were irrelevant, did not improve the care they provided or were purely a tick box exercise. Participants also felt irritated that there were errors in some of the information provided.

> *But I know that a lot of those warnings are wrong. They're just simply wrong.*
> [Participant No:2 GP Partner, Male, Y&H]

Participants considered some reminders were too sensitive, for example, recording hay fever in the patient's notes could trigger a sepsis alert. In some cases over sensitive reminders were removed.

For peer review only - http://bmjopen.bmj.com/site/about/guidelines.xhtml

Yeah, it's gone, I think it was flawed. It was coming up on virtually everything so I don't know who decided to switch that off

[Participant No:9 Nurse Practitioner, Female, Y&H]

Inappropriate timing of reminder

The reason for the patient visit

Participants questioned the appropriateness of the reminders and the need for opportunistic management during the consultation.

It's embarrassing, you've got a patient who's coming in who looks like they're in pain or stress and the first thing you have to do is ask about smoking

[Participant No:10 GP Partner, Female, NWL]

Within the consultation

Participants believed the timing of the alert could be made more appropriate to the consultations natural progression.

it would be nice if the reminder came up at the appropriate time, so if you are doing a history … there was a history based reminder, if the medication one came up in the medication, when you press examination maybe all the examination ones so that height, weight, blood pressure came up at that time so that, you know, you could do it in a more natural flowing

[Participant No:10 GP Partner, Female, NWL]

Participants felt that when reminders appeared when the clinician was not with a patient (e.g. drug interaction or drug alternative reminders when managing repeat prescriptions) this was annoying.

In comparison to the last reminder

Participants talked of how the time of year influenced when reminders appeared, for example Quality Outcomes Framework indicators are reset in April. Many felt algorithms could be developed so the reminder's timing was more meaningful and took into consideration when the previous reminder was actioned.

Changing systems

Participants spoke of frustration in the way systems constantly changed and that not enough precaution was taken to ensure new reminders were fully functional or user friendly.

For peer review only - http://bmjopen.bmj.com/site/about/guidelines.xhtml

Negative consequences of using reminders

Reminders are changing behaviour

Participants indicated that reminders have altered GP behaviour within the consultation. It was felt that GP training teaches patient centred care yet some reminders drive behaviour based on financial incentives. Participants felt they and others could become too reliant on reminders. There was also concern that reminders can force an inappropriate action, recording of incorrect information or make them wary of the information they recorded.

> *So, because the CCG [Clinical Commissioning Group] will be looking at our referral letters we should get patient's consent to allow them to look at it, that virtually never happens but you have to tick the box*
> [Participant No:8 GP Partner, Male, Y&H]

> *... they do change the way you do things. So I gave you the example of the sepsis reminders. It makes you wary of putting certain information in because you know it's going to prompt a reminder.*
> [Participant No:2 GP Partner, Male, Y&H]

Costs and benefits

The participants felt that the reminders suggesting alternatives to cut costs were apparent to the patient and damaged the relationship between patient and clinician. Several participants commented that the cost of the price comparison software probably outweighed any savings.

Increasing workload

Participants pointed out that the alternative drug prescribed were often unavailable, this required the patient to come back which increased their workload.

A need to improve the number and perceived value of reminders

Making the number of reminders more manageable

Most participants felt reducing the number of reminders was important. Participants outlined that numbers could be limited by keeping reminders relevant; by removing duplications, which occur when patients have more than one comorbid condition; by removing drug interactions or price comparison alerts for repeat prescriptions and joining up IT systems across the whole of health and social care to prevent duplication of work.

For peer review only - http://bmjopen.bmj.com/site/about/guidelines.xhtml

Where we're missing information is where things are done outside the practice so, you know, if we had information that was more freely available within the consultation that would prevent duplication, errors, all sorts of things

[Participant No:10 GP Partner, Female, NWL]

Some participants commented that reminders would be less frequent and more relevant if the algorithms used to trigger the reminders were more 'intelligent'.

Standardising and providing more context to a reminder

Participants felt that more context could be given to some reminders. For example, if a patient had previously been advised to have a smear test or a blood pressure measurement is requested because of a high reading at the last visit it would save time going through the notes but also duplication of work. Participants also felt that more use of reminder templates could standardise data recording and help the clinician work more systematically in a consultation.

Improving the design of reminders

Participants repeatedly called for an improvement in the ease of coding to avoid entering text information. Participants suggested the use of hierarchical drop-down lists, for example, with a sepsis alert, lists could speed entering data on the underlying condition.

Most participants did comment that the alert designs made some reminders harder to ignore than others (e.g. red colour coded reminders)

They flash and they're big, red angry ones.

[GP Partner, Male, Yorkshire & Humber]

However, the participants had become accustomed to the format of the reminders so some questioned the importance of a redesign. Colour coding or ranking reminders was felt to be too simplistic to highlight important reminders but helpful to define the type of reminder or where the reminder fitted into the progression of a consultation. Participants did feel that redesign of reminders needed to be more individualised to a clinician's consultation style.

I think, ideally, they should be individualised so people consult in different ways, so if I use the computer in a way that I'm not really entering much until the patient has left the room, they don't work for me in that way

[Participant No:8 GP Partner, Male, Y&H]

For peer review only - http://bmjopen.bmj.com/site/about/guidelines.xhtml

A need to improve the efficiency in managing reminders

Working as a team

The majority of participants felt that reminders were well managed across the practice team, although, some did feel that improvements could be made. For example, they suggested receptionists could be encouraged to offer longer appointments to patients with a high number of quality standard reminders or have dedicated staff to manage them.

> *It's so difficult to be an acute care clinician plus chronic disease manager at the same time, and that's where alerts sometimes probably get missed or not missed, but consciously avoided. So, an appropriate kind of management of the resource in terms of thinking, right, do we need to have more acute care clinicians or chronic disease managers?*
> [GP Partner, Male, NW London]

Training

All participants commented that new starters, within their practice, received training on using the clinical systems. However, participants felt that the provision of training was variable and many participants felt they needed more training to increase efficiency and to standardise data entry, particularly when new reminders were introduced.

> *To be honest with you, I think we could all do with training. Like when they update, things like that, I think it would be nice to have a session to say there are recent updates, can all the doctors come and catch up?*
> [GP Partner, Female, NW London]

Patient self-management of conditions and data

Participants believed that patients' needed to take more responsibility for their own health. They indicated that managing reminders often required signposting patients, and believed that if patients were more aware of their own needs, they could organise their own care. Some participants also believed that patients' access to amend records would have a positive impact on the number of reminders as updating information (e.g. smoking status, QOF reminder) or organising their annual reviews would reduce numbers. Participants also felt that patients would become more aware of drug cost-savings and agree to switch to a cheaper brand if the reminders were available to patients.

For peer review only - http://bmjopen.bmj.com/site/about/guidelines.xhtml

Discussion

Key findings

Participants were familiar with the reminders in their clinical systems and felt many were important, providing up to date, evidence-based information. Participants' emotional response to the appearance of a reminder depended on the reminder and the situation. Factors influencing the decision to act on the information provided in a reminder related to situational factors (time constraints, appropriateness, reminder sensitivity) and to individual clinician factors (perceived value of advice, GP experience, consultation style). There were negative consequences of using reminders. For example, concerns were reported in becoming over-reliant on the reminders or being forced to record incorrect information. GPs and PNs views on improving reminders were related to firstly a need to improve the efficiency of managing reminders and secondly a need to reduce their number and improve their value.

Comparison with past research

Our study found similar barriers compared with previous studies.[7-10] These were time pressures in primary care; irrelevant or inappropriate alerts within the clinical workflow; a lack of standardisation of reminders and a need for training to effectively manage the reminders. Clinicians interviewed in England particularly about quality standard reminders,[20] discussed the loss of continuity of care and the increase in workload, but enablers were also reported including more job satisfaction and improved disease management. A study investigating attitudes and motivation of GPs and PNs to deliver public health programmes, found attitudes towards the reminders were mixed. Some participants in the study thought reminders were bothersome, irritating, and many switched them off; but others felt they facilitated a consultation and the structured templates ensured essential clinical information was documented.[21] A systematic meta-review (2015),[22] of eleven systematic reviews, suggested four significant challenges that needed to be addressed before clinical decision support systems (which includes diagnosis alerts) could fully support the diagnosis. The systems needed to 1) be adaptable and incorporate new knowledge; 2) apply a standardised approach; 3) be deeply integrated with the electronic health record to trigger appropriate timing of the reminder; 4) and have an understanding of the cognitive workflow of the user. Interestingly these findings from using a meta-review methodology closely relate to findings from our qualitative research.

For peer review only - http://bmjopen.bmj.com/site/about/guidelines.xhtml

Limitations

In-depth qualitative interviews can offer novel and varied insights into the thoughts and behaviour of participants. We have collected views and experiences from GP and PNs in varying positions based in practices of two regions (North West London, and Yorkshire and Humber) with varying demographic populations. We, therefore, feel our findings provide a working hypothesis that is transferable across all GPs and PNs within the English NHS. However, we do acknowledge that parts of the knowledge 'map' may be missing.

We did not interview practice managers or receptionists who may also be involved in managing reminders within the practice, however, our study focused on obtaining clinician's attitudes to reminders that are specifically delivered at the point of care. We also did not include practice-based pharmacists, who are increasingly being employed in GP surgeries to help to manage long-term conditions or provide advise to patients on multiple medicines. All participants were from practices using either EMIS Web or SystmOne administrative systems. These are the market leaders, however, 10% of practices in England do not use these systems.[23]

We acknowledge that bias could be introduced during the process of data collection, analysis or interpretation. We took steps to limit participant bias by ensuring anonymity and interviewing in a place that was acceptable to the participant. We limited researcher bias by using a peer-reviewed topic guide; having two researchers [LD and RM] to validate coding and co-develop the thematic map; we also involving all authors and a patient representative in interpreting the study findings.

Implications for future development and policy

Studies have discussed the effectiveness of 'modal' alerts in prescribing,[24] these are reminders where the system becomes unobtainable behind the alert until actions have been completed, forcing the user to input information. However, our participants found these reminders could be the most irritating, particularly if the information they provided was irrelevant or incorrect. Concerns that incorrect information was often being recorded when an action was forced or that users were altering their behaviour to avoid triggering these reminders, is worrying and needs addressing. However, these types of reminders do have their place if well designed, using 'intelligent' algorithms, and users have appropriate training.

A randomised controlled trial, using computer-simulated patients, found early diagnostic suggestions can improve the accuracy of GPs diagnoses.[25] Our participants commented that

For peer review only - http://bmjopen.bmj.com/site/about/guidelines.xhtml

processing the information provided by a patient during a consultation could become difficult during busy surgeries and considered reminders to be a useful tool in preventing missed diagnoses.

Our study highlights the need to improve the experience of GP and PNs when interacting with reminders in their patients' electronic health records. Sophisticated use of data to create the reminders; connecting IT systems across the whole of health and social care; and patients' access to their data, have all been highlighted as important to progress with the development and improvement of reminders in patient records. The Government's target to go paperless[26] and introduce a comprehensive system of electronic health records in England by 2020,[27] is a positive move forward in this process. Future development will also need to address different consulting styles used by GP and PNs who will require training to improve the efficiency of managing reminders.

Conclusions

GPs and PNs believe reminders are useful in supporting the provision of good quality care. Improving GPs and PNs' engagement with reminders centres on further developing their relevance to a GPs clinical practice which is personalised, considers cognitive workflow and suppresses inappropriate presentation.

For peer review only

References

1. Department of Health. *High Quality Care For All: NHS Stage Review Final Report*; 2008.
2. Shojania KG, Jennings A, Mayhew A, Ramsay C, Eccles M, Grimshaw J. Effect of point-of-care computer reminders on physician behaviour: a systematic review. *CMAJ* 2010;**182**:E216-25. https://doi.org/10.1503/cmaj.090578
3. Croskerry P. A universal model of diagnostic reasoning. *Academic medicine : journal of the Association of American Medical Colleges* 2009;**84**:1022-8. https://doi.org/10.1097/ACM.0b013e3181ace703
4. van der Sijs H, Aarts J, Vulto A, Berg M. Overriding of drug safety alerts in computerized physician order entry. *J Am Med Inform Assoc* 2006;**13**:138-47. https://doi.org/10.1197/jamia.M1809
5. Magnus D, Rodgers S, Avery AJ. GPs' views on computerized drug interaction alerts: questionnaire survey. *Journal of Clinical Pharmacy and Therapeutics* 2002;**27**:377-82. https://doi.org/10.1046/j.1365-2710.2002.00434.x
6. Zazove P, Plegue MA, Uhlmann WR, Ruffin MTt. Prompting Primary Care Providers about Increased Patient Risk As a Result of Family History: Does It Work? *J Am Board Fam Med* 2015;**28**:334-42. https://doi.org/10.3122/jabfm.2015.03.140149
7. Zazove P, McKee M, Schleicher L, Green L, Kileny P, Rapai M*, et al.* To act or not to act: responses to electronic health record prompts by family medicine clinicians. *J Am Med Inform Assoc* 2017;**24**:275-80. https://doi.org/10.1093/jamia/ocw178
8. Koskela T, Sandstrom S, Makinen J, Liira H. User perspectives on an electronic decision-support tool performing comprehensive medication reviews - a focus group study with physicians and nurses. *BMC Med Inform Decis Mak* 2016;**16**:6. https://doi.org/10.1186/s12911-016-0245-z
9. Lugtenberg M, Pasveer D, van der Weijden T, Westert GP, Kool RB. Exposure to and experiences with a computerized decision support intervention in primary care: results from a process evaluation. *BMC Fam Pract* 2015;**16**:141. https://doi.org/10.1186/s12875-015-0364-0
10. Medlock S, Eslami S, Askari M, Brouwer HJ, van Weert HC, de Rooij SE*, et al.* Attitudes and experience of Dutch general practitioners regarding computerized clinical decision support. *Stud Health Technol Inform* 2013;**186**:56-60.
11. Kersting C, Weltermann B. Electronic reminders to facilitate longitudinal care: a mixed-methods study in general practices. *BMC Med Inform Decis Mak* 2016;**16**:148. https://doi.org/10.1186/s12911-016-0387-z
12. Dickfos M, King D, Parekh S, Boyle FM, Vandelanotte C. General practitioners' perceptions of and involvement in health behaviour change: can computer-tailored interventions help? *Prim Health Care Res Dev* 2015;**16**:316-21. https://doi.org/10.1017/s1463423614000498
13. European Observatory on Health Systems and Policies. *The Health Systems and Policy Monitor*. URL: https://www.hspm.org/searchandcompare.aspx (accessed 24 November 2019).
14. Bryan C, Boren SA. The use and effectiveness of electronic clinical decision support tools in the ambulatory/primary care setting: a systematic review of the literature. *Inform Prim Care* 2008;**16**:79-91.
15. Jenssen BP, Bryant-Stephens T, Leone FT, Grundmeier RW, Fiks AG. Clinical Decision Support Tool for Parental Tobacco Treatment in Primary Care. *Pediatrics* 2016;**137**. https://doi.org/10.1542/peds.2015-4185
16. National Institute for Health Research. *Local Clinical Research Networks*. URL: https://www.nihr.ac.uk/nihr-in-your-area/local-clinical-research-networks.htm (accessed 18 Jan 2019).
17. Ajzen I. The theory of planned behavior. *Organizational Behavior and Human Decision Processes* 1991;**50**:179-211. https://doi.org/https://doi.org/10.1016/0749-5978(91)90020-T

For peer review only - http://bmjopen.bmj.com/site/about/guidelines.xhtml

18. Michie S, Richardson M, Johnston M, Abraham C, Francis J, Hardeman W, *et al.* The behavior change technique taxonomy (v1) of 93 hierarchically clustered techniques: building an international consensus for the reporting of behavior change interventions. *Ann Behav Med* 2013;**46**:81-95. https://doi.org/10.1007/s12160-013-9486-6

19. Braun V, Clarke V. Using thematic analysis in psychology. *Qualitative Research in Psychology* 2006;**3**:77-101. https://doi.org/10.1191/1478088706qp063oa

20. Maisey S, Steel N, Marsh R, Gillam S, Fleetcroft R, Howe A. Effects of payment for performance in primary care: Qualitative interview study. *Journal of Health Services Research & Policy* 2008;**13**:133-9. https://doi.org/10.1258/jhsrp.2008.007118

21. Ma RMMN. *What strategies influence general practitioners to deliver public health programmes? An example using the National Chlamydia Screening Programme*: London School of Hygiene and Tropical Medicine; 2017. https://doi.org/https://doi.org/10.17037/PUBS.04645512

22. Nurek M, Kostopoulou O, Delaney BC, Esmail A. Reducing diagnostic errors in primary care. A systematic meta-review of computerized diagnostic decision support systems by the LINNEAUS collaboration on patient safety in primary care. *European Journal of General Practice* 2015;**21**:8-13. https://doi.org/10.3109/13814788.2015.1043123

23. Kontopantelis E, Stevens RJ, Helms PJ, Edwards D, Doran T, Ashcroft DM. Spatial distribution of clinical computer systems in primary care in England in 2016 and implications for primary care electronic medical record databases: a cross-sectional population study. *BMJ Open* 2018;**8**:e020738. https://doi.org/10.1136/bmjopen-2017-020738

24. Scott GPT, Shah P, Wyatt JC, Makubate B, Cross FW. Making electronic prescribing alerts more effective: scenario-based experimental study in junior doctors. *Journal of the American Medical Informatics Association* 2011;**18**:789-98. https://doi.org/10.1136/amiajnl-2011-000199

25. Kostopoulou O, Rosen A, Round T, Wright E, Douiri A, Delaney B. Early diagnostic suggestions improve accuracy of GPs: a randomised controlled trial using computer-simulated patients. *Br J Gen Pract* 2015;**65**:e49-54. https://doi.org/10.3399/bjgp15X683161

26. NHS England. *Five Year Forward View*; 2014.

27. Kelsey T, Cavendish W. *Personalised Health and Care 2020: A Framework for Action* National Information Board, Department of Health and Social Care,.

For peer review only

For peer review only - http://bmjopen.bmj.com/site/about/guidelines.xhtml

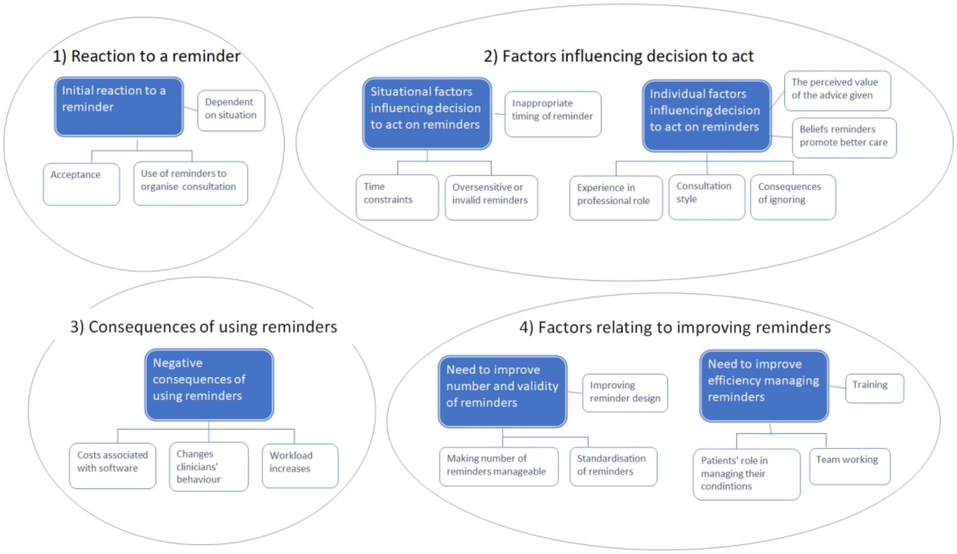

Figure 1: Thematic map of general practitioner and practice nurse views of electronic reminders

621x347mm (72 x 72 DPI)

Version no: 1 19.01.18

Imperial College London

Participant information sheet

Title of Project: Evaluating reminders in primary care electronic patient records

IRAS No: 234951

You are being invited to take part in a research study to evaluate GPs and practice nurses' attitudes towards reminders in electronic patient records. Before you decide it is important for you to understand why the research is being done and what it will involve. Please take time to read the following information carefully and discuss it with others if you wish.
Ask us if there is anything that is not clear or if you would like more information. Take time to decide whether or not you wish to take part.
Thank you for reading this.

Who will conduct the research?

This research is being conducted by Dr Elizabeth Cecil (Senior Researcher) and Professor Paul Aylin (Chief Investigator). The study is part of the Imperial Patient Safety Translational Research Centre's scheme of research. Development of the research has been supported and guided by a group clinicians, patients and researchers with an interest in primary care patient safety.

What is the purpose of the study?

Primary care administrative systems offer opportunity to improve patient care in the form of delivering reminders to primary health care professionals. An electronic reminder is a pop-up prompt created within a clinical administrative system at the point of care. These reminders remind a primary health care professional of an action required to provide good quality care or prevent harm to the patient. Examples of reminders are those that recommend: safe prescribing of medications (and warning of possible interactions); to provide advice on a preventive or clinical intervention (such as smoking cessation or immunisation); or to document clinical information (such as blood pressure). Attitudes to and the impact of reminders have been studied in the US, but little is known about attitudes in the UK. This research will inform further projects within Patient Safety and Translation Research Centre's programme of work to improve administrative systems, which will ultimately contribute to a safer NHS.

Why have I been chosen?

You have been chosen because as a primary health care professional you have extensive experience of using a primary care administrative system and therefore will have important insights into their electronic reminders. For this study we expect to recruit up to 25 further primary care nurses and GPs including partners and salaried GPs who will help us to get a rounded picture of the attitudes towards electronic reminders in primary care.

Do I have to take part?

No. It is up to you to decide whether or not to take part. If you do decide to take part you will be given this information sheet to keep and be asked to sign a consent form. If you decide to take part you are still free to withdraw at any time and without giving a reason.

What will happen to me if I take part?

If you decide to take part you will be asked a series of open ended questions to assess your views on reminders, which will be recorded for analysis. These questions will asked in a single interview, which can take place in a setting of your choice and will take a maximum of 1hr of your time (including the brief, consent and debrief). You will be reimbursed for your time and travel.

For peer review only

Version no: 1 19.01.18

What if something goes wrong?
If you are harmed by taking part in this research project, there are no special compensation arrangements. If you are harmed due to someone's negligence, then you may have grounds for a legal action. Regardless of this, if you wish to complain, or have any concerns about any aspect of the way you have been treated during the course of this study then you should immediately inform the senior researcher (e.cecil@imperial.ac.uk). The normal National Health Service complaints mechanisms are also available to you. If you are still not satisfied with the response, you may contact the Imperial AHSC Joint Research Compliance Office.

What are we studying
We are investigating attitudes towards reminders in primary care administrative systems. A reminder is a pop-up prompt created within a clinical administrative system at the point of care. These reminders remind a primary health care professional of an action required to provide good quality care or prevent harm to the patient. Examples of reminders are those that recommend: safe prescribing of medications (and warning of possible interactions); to provide advice on a preventive or clinical intervention (such as smoking cessation or immunisation); or to document clinical information (such as blood pressure).

What are the possible disadvantages of taking part?
We know that as a practising GP/Nurse your time is a limited. We will do our utmost not to inconvenience you and will ask for only 45 minutes of your time.

What are the possible benefits of taking part?
We cannot promise the study will directly affect your relationship with reminders in primary care, but the information could help to improve clinical systems which will ultimately contribute to a safer NHS.

Will my taking part in this study be kept confidential?
All information collected in both the consent form and the interview data will be kept strictly confidential. You will be assigned a code and only the senior researcher will have the key to identify participants. Personal data contained within the consent form will only be available to direct members of the study team and will be stored within the restricted secured networked IT system at Imperial College for 10 years after the completion of the study. The audio recordings of the interviews will be transcribed by a transcription company, Page Six, who have a confidentiality agreement in place with Imperial College London. The original recordings will be destroyed after transcription. Our procedures for handling, processing, storage and destruction of their data are compliant with the Data Protection Act 1998.

What will happen to the results of the research study?
Interviews will be anonymised using thematic analysis. Results will be written up into reports and research articles. These will include anonymised quotes from interviews. You will be notified of these before publication.

Who is organising and funding the research?
This study is part of the work of the Patient Safety and Translational Research Centre at Imperial College London (PSTRC). PSTRC is funded by the National Institute for Health Research.

Who has reviewed the study?
This study was reviewed by members of the PSTRC Research Partnership Group which comprises of patients and members of the general public, on 19.12.17. The Study Coordination Centre has obtained approval from the Health Regulator Authority (HRA).

Who should I contact for further information?
The senior researcher, Elizabeth Cecil can be contacted at:

e.cecil@imperial.ac.uk
020 7594 1838

Imperial College London,
Department of Primary Care and Public Health, Reynolds Building, St Dunstan's Road
London W6 8RP

For peer review only

Version no: 1 19.01.18

Imperial College London

Topic Guide

Title of Project: Evaluating reminders in primary care electronic patient records

IRAS No: 234951

Design: Semi-structured interview

Objectives:

- To assess GPs and practice nurses' familiarity with reminders in electronic health records.
- To determine GPs and practice nurses' views on the relevance of information provided by reminders in electronic health records.
- To understand the factors involved in why GPs and practice nurses ignore some reminders and not others.
- To explore how GPs and practice nurses feel reminders can be improved.

Brief:

Thank you very much for taking part in this study. My name is Elizabeth Cecil I work at Imperial College London. As you know we are carrying out a project to assess the primary health care professionals' views on reminders in electronic health records in primary care.

An electronic reminder is a pop-up prompt created within a clinical administrative system at the point of care. These reminders remind a primary health care professional of an action required to provide good quality care or prevent harm to the patient. Examples of reminders are those that recommend: safe prescribing of medications (and warning of possible interactions); to provide advice on a preventive or clinical intervention (such as smoking cessation or immunisation); or to document clinical information (such as blood pressure).

The interview is very informal and confidential. Only me and the direct members of my team will see or hear it. Your name will not appear on anything that we write and you will be notified before we publish the results. With your permission I would like to record the interview. This so that I can concentrate on your responses rather be distracted by taking notes. All recordings will be destroyed once the project is complete. Right, we'll both sign the two consent forms and there is a copy for you and one for me. Thank you we'll start. *Start taping.*

For peer review only

Version no: 1 19.01.18

Questions	Possible probes
**I would like to start by collecting some information on you and the practice**	
Please confirm your participant ID	It's written on your consent form
What is your position at this practice?	Are you a salaried GP, GP partner or practice nurse?
How long have you been qualified?	How long as a GP/practice nurse
What EHR system is used at this practice?	
Do you feel confident using the EHR system in your clinical practice?	Compared to your colleagues how competent do you feel you are?
**I now wish to discuss your familiarity with electronic reminders (knowledge)**	

For this research we define a reminder (in electronic primary care patient records) as:
A prompt created within a clinical administrative system at the point of care which reminds a primary health care professional of an required or desired action.

Can you give me some examples of reminders that you come across in your working day?	Are these the most common reminders
How many reminders do you tend to come across in a single day?	What factors are related to the number of reminders? E.g. time of year, patient age, comorbidities
Do reminders come up at other times than at the point of care, for example when the GP or administrator are adding patient information into the EHR?	Who is ultimately responsible for the outcome of a reminder if it does not always appear at point of care?
**(Skills)**<br>Do you feel confident to effectively manage reminders?	Is the training the GPs receive in the surgery or in their training elsewhere?
Have you been given training on how to respond to reminders?	
**Now I wish to explore your attitudes to reminders (beliefs – self-standards)**	
What do you think about reminders?	
How credible or relevant do you find the information provided by reminders systems?	
Can you give me an example of a reminder that you feel is particularly useful/relevant?	Why are they useful?<br>How much do you rely on these prompts?
**(To explore behaviour towards reminders / intentions)**	(Emotional response)

For peer review only

What is your immediate reaction to a reminder when it appears on your screen?	How does this make you feel e.g (delight, stress, dread)
Are there some reminders you are more likely to override/ignore than other others?	Why do you override/ignore this reminder compared with other reminders?
Can you expand on what factors dictate you to override/ignore?	For example are you more likely to ignore at the end of a day/week
Are there some reminders you never override/ignore?	Can you expand on why?
How often do you override/ignore reminders?	How many would that be, say, out of 10?
**(Beliefs about consequences)**<br>What will happen if you ignore a reminder?	
Can you give any example of a negative outcome after ignoring an alert?<br>How was this handled and was anything learned after the event?	If not by you but by any of your colleagues?
**(Social influences)**<br>To what extent does practice management or your peers influence your response to reminders?	
**(Decision processes)**<br>Do you feel that reminders benefit the care you provide in the consultation?	Do the reminders help you with the decisions you make on treating the patient?
Do you think there is convincing evidence to support the use of reminders in electronic health records?	

You have given me a detailed description of your attitudes towards reminders.

Do you think your colleagues both within your practice and within the profession feel the same way?	What dictates why they feel differently about reminders?
**(Constraints)**<br>What additional resources do you feel could help you effectively respond to a reminder?	Time is an obvious constraint but are there others?

Lets discuss your opinions on how to improve reminders

How would you improve the reminders if you could?	What specific changes do you feel would help facilitate the use of electronic reminders?
Do you feel that a redesign should include a ranking system?	For example colour coding for importance.
What information do you feel needs to be included that may have previously been missed?	

For peer review only

Version no: 1 19.01.18

Do you think these improvements will change your behaviour to and attitudes towards reminders?	Will you be less likely to ignore a reminder?

The Patient Safety and Translational Research Centre is carrying out research identify determinants of deterioration in primary care patients using EHRs.

Do you think that there are already reminders that can help to identify deteriorating patients or those patients where a diagnoses has previously been missed?	If not a reminder what other tools do you use?
Do you think that a reminder to flag up a deteriorating patient would be important?	

Debrief

Thank you for taking part. Do you have any questions that you want to ask me? If you do need to get hold of me in the future, my contact details are on the information sheet.

For peer review only

Themes and subthemes	Examples	Participant
**Reaction to a reminder**		
Frustration	*So, that can be quite frustrating, especially if you're thinking, you should realise why I'm ignoring this after a while*	[Participant No:10 GP Partner, Female, NWL]
	you end up just clicking cancel 100 times, it just irritates you in the consultation	[Participant No:16 Salaried GP, Female, NWL]
Acceptance	*I think that at this stage it doesn't make me feel anything because it's there all the time. I think it's become fairly automatic that you glance at it and it depends which one of the many warnings it is.*	[Participant No:4 GP Partner, Male, Y&H]
Dependent on situation	*If it's a busy clinic, stress, because it's, like, 'Ugh.' If it looks like it's going to be a complicated patient and it's an out-of-hours reminder, then relief probably.*	[Participant No:9 Nurse Practitioner, Female, Y&H]
Use of reminders to organise consultation	*What I tend to do, and I think most doctors do this, is we kind of do a pre-consultation before the list. So then, I would go through my list, see what reminders come up, and think, okay, fine, supposing it's a child, I'll remember to talk to Mum about reminding about immunisations.*	[GP Partner, Female, NW London]
**Individual factors influencing decision to act on reminders**		
The perceived value of the advice given		
Up to date advice	*So, the guidance is very accurate and up-to-date and regularly, it's responsive with regards to the changes that are happening in the BNF to be updated*	[Participant No:14 GP Partner, Male, NWL]
Reminding of infrequently required care	*the reminders for cervical smears, because it's three years so some people just don't receive the letters; so promoting them …*	[Participant No:15 GP Partner, Male, NWL]
The value of reminders to organise work		
Beliefs that reminders promote better care		
For continuity of care	*I appreciate them, I think that they have definitely a use because I can't remember everything*	[Participant No:10 GP Partner, Female, NWL]
For possible diagnoses	*if there's a kind of brain fatigue of seeing so many patients, it's easy sometimes to record, but not necessarily process, what you are writing. That's in any field. So, you're writing, yes, heart rate*	[Participant No:14 GP Partner, Male, NWL]

For peer review only

	150, I know that's high but I haven't really thought about it. So, if the sepsis alert comes up, I think it's good because it also has made you think.	
Experience in professional role, eg. Clinical interests or years in clinician practice		
Beliefs of one's personal capabilities	*I mean, I'd be worried if a GP at this stage wasn't aware of sepsis*	[Participant No:16 Salaried GP, Female, NWL]
Beliefs of others confidence capabilities	*And I think doctors are more confident to ignore the prescribing advice than I would be at all*	[Participant No:13 Nurse Practitioner, Female, NWL]
Feelings of being in control	*I will try, like this morning, I've got quite a few I've done the whole lot of, and I feel really satisfied.*	[Participant No:13 Nurse Practitioner, Female, NWL]
Interests and belief in importance of reminder	*everyone's the same, their interests or their increased fields of knowledge are the ones that they're sort of going to look at more, so even if they are improved, things like the patients with rheumatoid arthritis and things that need reviews*	[Participant No:17 Nurse Practitioner, Female, NWL]
Position and importance of reminder	*I think the partners will really try and crack through the things more. One could argue it's because money is involved*	[Participant No:10 GP Partner, Female, NWL]
Feelings of responsibility	*Doctors …they might ignore the foot check whereas myself or my colleagues, peers, would either do it or tell somebody to go and book an appointment*	[Participant No:17 Nurse Practitioner, Female, NWL]
Feelings of guilt	*I will feel guilty, if I haven't asked somebody whether they smoke I feel a twinge of guilt, but clinically it's probably not going to do a great deal*	[Participant No:9 Nurse Practitioner, Female, Y&H]
Consultation style		
	Writing up oh I should have done that doesn't matter then whether I'm on time or 20 minutes late because they've still gone out the door.	[Participant No:3 GP Partner, Male, Y&H]
Consequences of ignoring reminders		
Litagation	*.... people are very reluctant to make a change because... nobody wants to take responsibility. Everybody's scared of being sued and the computer companies are very scared of being sued... so they overdo it. They absolutely overdo it. And unfortunately that therefore makes the warnings much weaker because they're not specific enough.*	[Participant No:2 GP Partner, Male, Y&H]
Litagation	*I'm very risk-averse, and I'm always aware of litigation.*	[Participant No:9 Nurse Practitioner, Female, Y&H]
Litagation	*I'm not convinced that the MDU [Medical Defence Union] would defend me in the same way as they would a GP,*	[Participant No:9 Nurse Practitioner, Female, Y&H]

For peer review only

Patient Harm	*The only negative outcome is if you ignored an important alert. A lot of them are about money, so ignoring … instruction to use a cheaper drug… you might get a rebuke….Obviously, if it's about patient safety - and a lot of them are about patient safety - but if it feels a true risk and you ignored it then the consequence is that you would do the patient harm.*	[Participant No:2 GP Partner, Male, Y&H]
Financial loss	*A lot of them are about money, so ignoring the CCG's instruction to use a cheaper drug, sometimes you might get a rebuke if it's a really, really expensive decision. But that would be as rare as hen's teeth that that would happen.*	[Participant No:2 GP Partner, Male, Y&H]
Lost opportunity	*example cervical screening, and I can't remember a case off the top of my head. But if you ignored it and then you didn't necessarily remind the patient and you think, this is a young person who doesn't really come into the surgery, this was an opportunistic consultation, it would have been a great chance to remind them of that,*	[Participant No:14 GP Partner, Male, NWL]
Increase workload	*Well ultimately, you have to call the patient back in to get the work done, so ultimately yes.*	[Participant No:13 Nurse Practitioner, Female, NWL]
Audit	*I mean I was told that they do audit who's doing the flus so who's choosing to say, no, come back but then they can look at that and see was it appropriate because obviously if they're unwell*	[Participant No:17 Nurse Practitioner, Female, NWL]
Genuine reasons for ignoring reminders	*I would defend – if I got picked up on it, and I wouldn't often – I would be able to defend why I ignored it for that patient.*	[Participant No:13 Nurse Practitioner, Female, NWL]
**Situational factors influencing decision to act on reminders**		
Time constraints	*I wouldn't be able to do anything if I didn't ignore the majority of them.*	[Participant No:2 GP Partner, Male, Y&H]
	That's pretty standard, I mean here they do 10 minutes and every hour you get a 10 minute block so they say that's 12 minutes.	[Participant No:17 Nurse Practitioner, Female, NWL]
Inappropriateness of reminder's appearance		
Within consultation flow	*I think they're fantastic if they're at the right time.*	[Participant No:8 GP Partner, Male, Y&H]
	And instead of them just being, for example, a box somewhere it would be nice if the reminder came up at the appropriate time, so if you are doing a history if the appropriate reminder came up in… there was a history based reminder, if the medication one came up in the medication, when you press examination maybe all the examination ones so that height, weight, blood pressure came up at that time so that, you know, you could do it in a more natural flowing	[Participant No:10 GP Partner, Female, NWL]
	Some of the other prompts tend to come afterwards rather than at the point of care,	[Participant No:8 GP Partner, Male, Y&H]
In relation to reason for patient visit	*It's embarrassing, you've got a patient who's coming in who looks like they're in pain or stress and the first thing you have to do is ask about smoking -*	[Participant No:10 GP Partner, Female, NWL]

In relation to the time of year	*I'm not going to respond to anything to do with the flu jab at this point in time because it's the wrong time of year.*	[Participant No:5 GP Partner, Female , NWL]
	with QOF related indicators there is a timeframe and, … there may be a reminder to do something that was only done two or three months ago, for which there is no real clinical need to do now but because the system is set up in that way..	[GP Partner, Male, NW London]
In timing since previous reminder	*And so do you think that a reminder should have a sort of additional algorithm that says basically six months afterwards*	[Participant No:12 GP Partner, Male, NWL]
Invalid reminders	*But I know that a lot of those warnings are wrong. They're just simply wrong.*	
Oversensitive reminders	*Extremely sensitive. So, if you put in something like hay fever, it will come up with a sepsis alert, because it will just catch that bit that you say fever, so a lot of time you end up just going exit, exit, exit and it actually creates more work. I don't think my consultations have ever benefitted from a sepsis alert, never.*	[Participant No:16 Salaried GP, Female, NWL]
Oversensitive reminders	*Yeah, it's gone, I think it was flawed. It was coming up on virtually everything so I don't know who decided to switch that off*	[Participant No:9 Nurse Practitioner, Female, Y&H]
System changes	*So, for example, atrial fibrillation has a new template that's come out on the EMIS and I just had a patient today with atrial fibrillation and I noticed it wasn't working properly. So then, I would say to – I rang the manager to say, "Can we just go through this at one point?" Because I think if I'm having problems with it, we probably are as a group because it doesn't seem to be uploaded properly.*	[Participant No:11 GP Partner, Female, NWL]
**Negative concequences of using reminders**		
Costs associated with software outway any benefits	*The original people that did the alerts were appalling and there was evidence that the cost of the alerts was way in excess of the savings …. So there's lots of examples in the NHS where we make really poor financial decisions. Popups can be one of those.*	[Participant No:2 GP Partner, Male, Y&H]
Permanency of data recorded	*And I know other patients, even a doctor colleague who had something put on her child's records because she was widowed and so the child was considered more vulnerable. And fair enough, so somebody else looking out for them and thinking about their needs, but it was put on in such a way that it looked like the child was in need on a protection plan, you know, being mishandled. And she couldn't get it taken off for love nor money even though she was a perfectly competent parent and there was a perfectly reasonable need in that child being more vulnerable because her child's father had just died. So there are pop-ups that can be damaging, actually.*	[Participant No:5 GP Partner, Female , NWL]
Changes PHCPs behaviour		

For peer review only

Changes in the way data is recorded	*they do change the way you do things. So I gave you the example of the sepsis reminders. It makes you wary of putting certain information in because you know it's going to prompt a reminder.*	[Participant No:2 GP Partner, Male, Y&H]
Distracts PHCP from patient centredness	*they've just got to be careful that they're not too much of a burden and too intrusive all the time, because it then does detract you away from your primary objective, which is, kind of, interacting with that patient,*	[Participant No:4 GP Partner, Male, Y&H]
Patients aware reminders impact on behaviour		
Damages patient-PHCP relationship	*But a lot of patients also have an awareness that it earns us money. And they will quote that back at you. So, you have to be careful and you have to, you know… I think…*	[Participant No:9 Nurse Practitioner, Female, Y&H]
Workload increases as patients consult more	*and it's exactly the same medication just a different brand, so then you're spending more time explaining to them, "It's exactly the same thing". So, as I said, I appreciate why it's there; sometimes I do switch from the generic brand to the branded because it's whatever is in fashion and it's common, but there are*	[Participant No:15 GP Partner, Male, NWL]
Learning from mistakes	*It's not about blame. How can we avoid this? And we put strategies*	[Participant No:9 Nurse Practitioner, Female, Y&H]
Reminders force incorrect data entry	*So, because the CCG will be looking at our referral letters we should get patient's consent to allow them to look at it, that virtually never happens but you have to tick the box*	[Participant No:8 GP Partner, Male, Y&H]
**Need to improve number and validity of reminders**		
Making number of reminders more manageable		
Make all reminders relevant	I don't think anybody's ever complained about the serious alerts, it's the infuriating pop ups.	[Participant No:10 GP Partner, Female, NWL]
Make all reminders valid	So, I think if you say proceed, then it doesn't ask anything, but if you say ignore, it comes up saying, basically, why are you ignoring me? So, that can be quite frustrating, especially if you're thinking, you should realise why I'm ignoring this after a while	[Participant No:10 GP Partner, Female, NWL]
Make reminders more timely	with QOF related indicators there is a timeframe and, as I said, there may be a reminder to do something that was only done two or three months ago, for which there is no real clinical need to do now but because the system is set up in that way..	[Participant No:12 GP Partner, Male, NWL]
Joining up IT systems across health and social care	where we're missing information is where things are done outside the practice so, you know, if we had information that was more freely available within the consultation that would prevent duplication, errors, all sorts of things	[Participant No:10 GP Partner, Female, NWL]
Intelligent algorithms	*It's a bit annoying because something like that a lot of the alerts will come up if there's ever been a problem so you look back and you think oh I might actually ask the patient saying about your,*	[Participant No:17 Nurse Practitioner, Female, NWL]

For peer review only

	you know, liver function and they'll go, 'Oh that was four years ago and it's all fine now and I used to drink and I don't anymore' but EMIS… I don't know how far back EMIS goes but it seems to pick up anything abnormal but you have to check it.	
Make reminders more timely	*with QOF related indicators there is a timeframe and, as I said, there may be a reminder to do something that was only done two or three months ago, for which there is no real clinical need to do now but because the system is set up in that way..*	[Participant No:12 GP Partner, Male, NWL]
Improving the evidence base of reminders	*QOF, the evidence-base for that is poor, you know, not robust. And I expect that the evidence-base for this will be mixed. But I don't think it's going to mean that they go away.*	[Participant No:9 Nurse Practitioner, Female, Y&H]
Standardisation of reminders	*I'd like an NHS formulary that was set nationally*	[Participant No:10 GP Partner, Female, NWL]
Providing more context to a reminder	*let's say I saw a patient and they've got a whole list of things that need actioning; if someone's seen them two days before I don't know whether they have addressed any of that, whether they have highlighted, "Go for your asthma review". So, that's why it's always really useful in the notes to put, "Verbal invite given for smear", so you know actually the person two days ago when they saw them for whatever reason has prompted them to go for the smear.*	[Participant No:15 GP Partner, Male, NWL]
Need to improve the design of reminders		
Use of templates with drop down menus	*and then you could actually have something saying it's an ENT, it's a chest, it's an acute abdomen, it's a urine, you know, and then you could just quickly pick which one you want and then you could put that information in because the advantages of that is that those would be just a tick box exercise whereas when you add it yourself you would put in urine analysis then you have a drop down then you have to find it, you know? So, you could just free text it but that's ridiculous, you don't want to free text anything, you want to read code as much as you possibly can.*	[Participant No:10 GP Partner, Female, NWL]
Ranking in reminders isn't easy	*Ranking on what basis? It's hard to know whether a patient's allergy is more important than a child's protection need or a patient's, you know, medication review*	[Participant No:5 GP Partner, Female , NWL]
Personalised	*I think, ideally, they should be individualised so people consult in different ways, so if I use the computer in a way that I'm not really entering much until the patient has left the room, they don't work for me in that way*	[Participant No:8 GP Partner, Male, Y&H]
Better use of icons	*They could be personalised, not quite as funkily as this where it's very obvious to me within a flash that this patient smokes - she uses an inhaler and she's probably got restrictive or obstructive or interstitial lung disease - that she's got brittle bones, well she's definitely got lung disease, that she's at risk for dementia because (overspeaking), that she might not have had her flu jab. I*	[Participant No:5 GP Partner, Female , NWL]

For peer review only

	mean, I can tell very, very quickly. I never need to ask somebody whether they smoke or not; I just look at the screen...So the icons are quite useful in that respect.	
Colour coding	*actually to be able to look at a screen and go the clinical, the actions one – i.e. blood pressure, BMI – coming up as one colour and the more talky ones coming up as a different one would actually probably really help because then you could go, 'Oh, there's only three things I need to actually do, I just need to ask them a few questions'.*	[Participant No:13 Nurse Practitioner, Female, NWL]
**Need to improve efficiency managing reminders**		
Working as a team		
Training	*… no, sometimes extra training to know what's the quickest way of doing something, …., so if it's recording, say, a vaccination that you've given, it might say that they need a B12 is it quicker to use templates?*	[Participant No:17 Nurse Practitioner, Female, NWL]
Need to change attitudes	*I think the alerts are just going to be annoying going forward until we as clinicians change the way that we have to input data*	[Participant No:9 Nurse Practitioner, Female, Y&H]
Reorganisation of chronic disease care	*At the moment, there's a real kind of shift in what a lot of GPs are doing at the moment, so it's moved from, historically, a lot of chronic disease management and now it's a lot of acute care. It's getting increasingly difficult to be both kinds of doctor in the ten minutes. So, to respond to reminders, in theory, for the best interests of patients, you've got to be thinking, can chronic disease management be managed in a different way with stable patients, it's like almost a chronic disease birthday or something like that where the patient thinks, right, every year in the month of my birthday, the practice is going to deal with all my reminders to do with diabetes or whatever chronic disease it is. It's so difficult to be an acute care clinician plus chronic disease manager at the same time, and that's where alerts sometimes probably get missed or not missed, but consciously avoided. So, an appropriate kind of management of the resource in terms of thinking, right, do we need to have more acute care clinicians or chronic disease managers?*	[Participant No:14 GP Partner, Male, NWL]
Patient self management	*And even if it's, "This patient needs to book an appointment" – the patient needs to take responsibility as well for their own health; you can't hand-hold everybody. But even something that has said, "A message, a text, or a call, or an appointment, has been sent to this patient to discuss their blood pressure", would be useful.*	[Participant No:15 GP Partner, Male, NWL]

For peer review only

COREQ (COnsolidated criteria for REporting Qualitative research) Checklist

A checklist of items that should be included in reports of qualitative research. You must report the page number in your manuscript where you consider each of the items listed in this checklist. If you have not included this information, either revise your manuscript accordingly before submitting or note N/A.

Topic	Item No.	Guide Questions/Description	Reported on Page No.
**Domain 1: Research team and reflexivity**			
Personal characteristics			
Interviewer/facilitator	1	Which author/s conducted the interview or focus group?	
Credentials	2	What were the researcher's credentials? E.g. PhD, MD	
Occupation	3	What was their occupation at the time of the study?	
Gender	4	Was the researcher male or female?	
Experience and training	5	What experience or training did the researcher have?	
Relationship with participants			
Relationship established	6	Was a relationship established prior to study commencement?	
Participant knowledge of the interviewer	7	What did the participants know about the researcher? e.g. personal goals, reasons for doing the research	
Interviewer characteristics	8	What characteristics were reported about the inter viewer/facilitator? e.g. Bias, assumptions, reasons and interests in the research topic	
**Domain 2: Study design**			
Theoretical framework			
Methodological orientation and Theory	9	What methodological orientation was stated to underpin the study? e.g. grounded theory, discourse analysis, ethnography, phenomenology, content analysis	
Participant selection			
Sampling	10	How were participants selected? e.g. purposive, convenience, consecutive, snowball	
Method of approach	11	How were participants approached? e.g. face-to-face, telephone, mail, email	
Sample size	12	How many participants were in the study?	
Non-participation	13	How many people refused to participate or dropped out? Reasons?	
Setting			
Setting of data collection	14	Where was the data collected? e.g. home, clinic, workplace	
Presence of non-participants	15	Was anyone else present besides the participants and researchers?	
Description of sample	16	What are the important characteristics of the sample? e.g. demographic data, date	
Data collection			
Interview guide	17	Were questions, prompts, guides provided by the authors? Was it pilot tested?	
Repeat interviews	18	Were repeat inter views carried out? If yes, how many?	
Audio/visual recording	19	Did the research use audio or visual recording to collect the data?	
Field notes	20	Were field notes made during and/or after the inter view or focus group?	
Duration	21	What was the duration of the inter views or focus group?	
Data saturation	22	Was data saturation discussed?	
Transcripts returned	23	Were transcripts returned to participants for comment and/or	

For peer review only

Topic	Item No.	Guide Questions/Description	Reported on Page No.
		correction?	
**Domain 3: analysis and findings**			
Data analysis			
Number of data coders	24	How many data coders coded the data?	
Description of the coding tree	25	Did authors provide a description of the coding tree?	
Derivation of themes	26	Were themes identified in advance or derived from the data?	
Software	27	What software, if applicable, was used to manage the data?	
Participant checking	28	Did participants provide feedback on the findings?	
Reporting			
Quotations presented	29	Were participant quotations presented to illustrate the themes/findings? Was each quotation identified? e.g. participant number	
Data and findings consistent	30	Was there consistency between the data presented and the findings?	
Clarity of major themes	31	Were major themes clearly presented in the findings?	
Clarity of minor themes	32	Is there a description of diverse cases or discussion of minor themes?	

Developed from: Tong A, Sainsbury P, Craig J. Consolidated criteria for reporting qualitative research (COREQ): a 32-item checklist for interviews and focus groups. *International Journal for Quality in Health Care*. 2007. Volume 19, Number 6: pp. 349 – 357

Once you have completed this checklist, please save a copy and upload it as part of your submission. DO NOT include this checklist as part of the main manuscript document. It must be uploaded as a separate file.

For peer review only

BMJ Open

General practitioner and nurse practitioner attitudes towards electronic reminders in primary care: A qualitative analysis

Journal:	*BMJ Open*
Manuscript ID	bmjopen-2020-045050.R1
Article Type:	Original research
Date Submitted by the Author:	14-Mar-2021

Complete List of Authors:	Cecil, E; Imperial College London, Primary Care and Public Health; King's College London, School of Life Course Sciences<br>Dewa, Lindsay; Imperial College London, School of Public Health; Imperial College London, Patient Safety Translational Research Centre<br>Ma, Richard; Imperial College London, Primary Care and Public Health<br>Majeed, Azeem; Imperial College London, Primary Care<br>Aylin, Paul; Imperial College London, Primary Care and Public Health

**Primary Subject Heading**:	Health services research
Secondary Subject Heading:	Qualitative research
Keywords:	PRIMARY CARE, Information technology < BIOTECHNOLOGY & BIOINFORMATICS, Quality in health care < HEALTH SERVICES ADMINISTRATION & MANAGEMENT

SCHOLARONE™
Manuscripts

I, the Submitting Author has the right to grant and does grant on behalf of all authors of the Work (as defined in the below author licence), an exclusive licence and/or a non-exclusive licence for contributions from authors who are: i) UK Crown employees; ii) where BMJ has agreed a CC-BY licence shall apply, and/or iii) in accordance with the terms applicable for US Federal Government officers or employees acting as part of their official duties; on a worldwide, perpetual, irrevocable, royalty-free basis to BMJ Publishing Group Ltd ("BMJ") its licensees and where the relevant Journal is co-owned by BMJ to the co-owners of the Journal, to publish the Work in this journal and any other BMJ products and to exploit all rights, as set out in our licence.

The Submitting Author accepts and understands that any supply made under these terms is made by BMJ to the Submitting Author unless you are acting as an employee on behalf of your employer or a postgraduate student of an affiliated institution which is paying any applicable article publishing charge ("APC") for Open Access articles. Where the Submitting Author wishes to make the Work available on an Open Access basis (and intends to pay the relevant APC), the terms of reuse of such Open Access shall be governed by a Creative Commons licence – details of these licences and which Creative Commons licence will apply to this Work are set out in our licence referred to above.

Other than as permitted in any relevant BMJ Author's Self Archiving Policies, I confirm this Work has not been accepted for publication elsewhere, is not being considered for publication elsewhere and does not duplicate material already published. I confirm all authors consent to publication of this Work and authorise the granting of this licence.

For peer review only

General practitioner and nurse practitioner attitudes towards electronic reminders in primary care: A qualitative analysis

Elizabeth Cecil, Lindsay Dewa, Richard Ma, Azeem Majeed, Paul Aylin

The Dr Foster Unit, Department of Primary Care and Public Health, Imperial College London, 3 Dorset Rise, London EC4Y 8EN, UK
Elizabeth Cecil, Research Associate
Lindsay Dewa, Research Associate
Paul Aylin, Professor of Epidemiology and Public Health

Department of Primary Care and Public Health, Imperial College London, W6 8RP, UK
Richard Ma, NIHR Doctoral Research Fellow (Clinical)
Azeem Majeed, Professor of Primary Care

Correspondence to Elizabeth Cecil: e.cecil@imperial.ac.uk, +44 2075941838

Acknowledgements

We wish to thank Puja Verma, a GP in North West London, for her support developing the topic guide. To members of the Research Partners group for their contribution in the project development and result interpretation. A thank you to Sean Shillito, North West London CRN and Michelle Platton, Yorkshire and Humber CRN for their support in recruiting participants for this study.

Funding

This study was funded by National Institute for Health Research (NIHR) Patient Safety Translational Research Centre (PSTRC) programme grant (PSTRC-2016-004). RM is funded by a NIHR Doctoral Research Fellowship (DRF-2017-10-181) for this research project. The NIHR Imperial Patient Safety Translational Centre is a partnership between the Imperial College Healthcare NHS Trust and Imperial College London. The Dr Foster Unit is an academic unit in the Department of Primary Care and Public Health, within the School of Public Health, Imperial College London. The unit receives research funding from Dr Foster Intelligence, an independent health service research organisation (a wholly owned subsidiary of Telstra). The Department of Primary Care & Public Health at Imperial College London is grateful for support from the NW London NIHR Applied Research Collaboration and the Imperial NIHR Biomedical Research Centre. The views expressed in this publication are those of the authors and not necessarily those of the NHS, the NIHR, MRC, CCF, NETSCC, the HSR programme or the Department of Health.

Conflicts of interest

All authors have completed the Unified Competing Interest form (available on request from the corresponding author), and PA and AB declare that they are partially funded by grants from Dr Foster Intelligence, an independent healthcare information company.

For peer review only - http://bmjopen.bmj.com/site/about/guidelines.xhtml

Contributors

PA secured the funding for this study. EC, LD, RM, AM and PA contributed to the conception and design. EC secured Health Research Authority approval for the study and conducted all interviews. EC, LD and RM carried out the analysis. All authors (EC, LD, RM, AM and PA) took part in interpreting the findings. All authors commented on and helped to revise drafts of this paper. All authors have approved the final version.

Data availability

Deidentified participant data are available, from the corresponding author, upon reasonable request. Theme examples within the interview data are shown in supplementary file 2.

Ethics Approval

Health Research Authority (HRA) approval was gained (IRAS No: 234951). Ethics approval was not required for this study as participants were NHS staff.

For peer review only

For peer review only - http://bmjopen.bmj.com/site/about/guidelines.xhtml

Abstract

Objectives

Reminders in primary care administrative systems aim to help clinicians provide evidence-based care, prescribe safely and save money. However, increased use of reminders can lead to alert fatigue. Our study aimed to assess general practitioners' (GP) and nurse practitioners' (NP) views on electronic reminders in primary care.

Design

A qualitative analysis using semi-structured interviews.

Setting and Participants

Fifteen GPs and NP based in general practices located in North-West London and Yorkshire, England.

Methods

We collected data on participants' views on; i) perceptions of the value of information provided; ii) reminder-related behaviours and iii) how to improve reminders. We carried out a thematic analysis.

Results

Participants were familiar with reminders in their clinical systems and felt many were important to support their clinical work. However, participants reported, on average, 70% of reminders were ignored. Four major themes emerged. 1) Reaction to a reminder, which was mixed and varied by situation. 2) Factors influencing the decision to act on reminders, often related to experience, consultation styles, and interests of participants. Time constraints, alert design, inappropriate presentation, and litigation were also factors. 3) Negative consequences of using reminders were increased workload or costs, and compromising GP and NPs behaviour. 4) Factors relating to improving users' engagement with reminders, were prevention of unnecessary reminders through data linkage across healthcare administrative systems or the development of more intelligent algorithms. Participants felt training was vital to effectively manage reminders.

Conclusions

GPs and NPs believe reminders are useful in supporting the provision of good quality patient care. Improving GPs and NPs' engagement with reminders centres on further developing their relevance to their clinical practice which is personalised, considers cognitive workflow and suppresses inappropriate presentation.

For peer review only - http://bmjopen.bmj.com/site/about/guidelines.xhtml

Strengths and limitations of this study

- Our use of in-depth qualitative interviews has produced novel and varied insights into GP and nurse practitioners' attitudes toward reminders in electronic health records.
- The views and experiences we collected from GP and nurse practitioners in varying positions based in practices of two regions (North West London, and Yorkshire and Humber) with varying demographic populations has provided a working hypothesis that, we feel, is transferable across GP and nurse practitioners working within the English NHS.
- We limited researcher bias by using a peer-reviewed topic guide; having two researchers to validate coding and co-develop the thematic map.
- However, we do acknowledge that parts of the knowledge 'map' may be missing. Primary care clinicians who engaged with the study may have different views than those who did not.

For peer review only - http://bmjopen.bmj.com/site/about/guidelines.xhtml

Introduction

Good quality health care is safe, effective and patient centred.[1] Primary care administrative systems offer the opportunity to improve patient care by delivering reminders to clinicians at the point of care. An electronic reminder is a prompt created within a clinical administrative system, through the interrogation of recorded patient data. They remind the clinician of an action required, which may include a recommendation on safe medication prescribing (warning of possible interactions); a more cost-effective drug; advice on a preventive or clinical intervention (such as smoking cessation or immunisation); or to document clinical information (such as blood pressure) (Table 1).

There is evidence that clinician behaviours and processes of care provision can be improved by point of care computer reminders, but improvements tend to be small and the features of reminder systems that are associated with clinically worthwhile improvements are difficult to determine.[2] However, in primary care, reminders are believed to be particularly important. This is because within the consultation, time pressures, limited information and distracting cues mean that clinicians' thinking (general practitioners (GP) and nurse practitioners (NP)), needs to be fast and intuitive rather than systematic.[3] Consequently, there has been an increasing use of reminders for a range of clinical tasks (Table 1).

There are barriers to clinical engagement with the reminders. One such barrier is 'alert fatigue',[4] a potential unintended consequence of the increased number of reminders. Alert fatigue happens when the system user becomes desensitised to the digital reminders, and subsequently fails to engage with them. A literature review (predominantly from the USA and within hospital settings) that investigated reminders safety in medication prescribing, found between 49–96% of alerts were overridden.[4] In 2002 a UK cross-sectional study of primary care clinicians assessed attitudes towards drug interaction reminders and found 1 in 4 (of 236 responses) admitted to frequently overriding drug interaction alerts without properly checking them.[5] Other barriers include lack of user friendliness in the administrative systems; time constraints to address the reminder; and clinicians being unable to see the value in the information provided were all barriers to the acceptance of reminders.[6 7-10]

Most research in this area has been outside the UK,[8, 11, 12] where health systems, such as primary care gate-keeping role, can differ.[13] Whilst studies have examined the development of computerised systems to improve quality in primary care,[14, 15] studies tend to be quantitative. An up to date understanding on how GPs and NPs interact with these systems within the UK is lacking. This study aims to address this gap using semi-structured interviews to explore GP and NPs attitudes towards electronic reminders in primary care, to support their enhancement.

For peer review only - http://bmjopen.bmj.com/site/about/guidelines.xhtml

Table 1: Types of reminders in primary care clinical computer systems

Reminder type	Software	Purpose	Viewed	Action required	Modal*
**Drug price comparison alert**	Prescribing decision support software e.g. ScriptSwitch, OptimiseRx	To save on costs of prescribed drugs	A pop-up viewed when prescribing suggesting a cheaper alternative	Action is required to close the pop-up. Information can be ignored	✓
**Drug interaction/monitoring alert**	GP system standard template. Based on UK pharmaceutical reference publications Algorithm draws on patient prescription data	To promote safe prescribing	A pop-up viewed when prescribing warning of potential drug interactions	Action is required to close the pop-up. Information can be ignored	✓
**Allergy alert**	GP system standard template Algorithm draws on patient clinical data	To promote safe prescribing	A pop-up viewed when prescribing warning of potential allergies	Action is required to close the pop-up. Information can be ignored	✓
**Diagnosis alert**	Diagnosis decision support reminder e.g. Sepsis	To increase awareness of condition as a possible diagnosis	A pop-up viewed when entering diagnosis related patient symptoms	Data must be entered to close the pop-up	✓
**Quality care standard reminders (e.g.Quality Outcomes Framework, Flu vaccine)**	GP system templates and toolkits; Algorithms draw on patient clinical data	To promote evidence-based care and maximise financial payment for performance	On the patient home page and viewed when patient record is opened. Information continues to appears as icons when records are updated	Most reminders don't have to be actioned and can be ignored	X
**Additional patient information alerts from set reminder**	Personally created from set template e.g. Safeguarding children template (Royal College of General Practice)	To communicate patient specific information to other clinicians. To create a registry	On the patient home page and viewed when patient record is opened to those granted access	No action required when viewing reminder	X

For peer review only - http://bmjopen.bmj.com/site/about/guidelines.xhtml

**Additional patient information from generic reminder**	Personally created from a generic template or free text e.g. highlighting a patient with additional needs	To communicate patient specific information to oneself or to other clinicians and practice staff	On the patient home page and viewed when patient record is opened to those granted access	No action required when viewing reminder	X

* Modal alerts are those where the system shuts down and becomes unobtainable behind the alert. The user must interacted with the alert in order to return to the patient record.

For peer review only

Methods

Design

We conducted a qualitative study using semi-structured interviews. The use of semi-structured interviews was chosen because the methodology allows a flexibility to the interview process, which results in the collection of data relating to GP and NPs' attitudes to reminders in electronic health records.

Participants

We recruited a purposeful sample of GPs and NPs from NHS general practices located in two regions in England: North West London (NWL), and Yorkshire and Humber (Y&H) between 12th March and 19th September 2018. These regions were selected to obtain opinions from participants serving populations with varying demographics (e.g. rural compared with urban patients). We selected participants with experience of primary care administrative systems, based on their position, experience and practice population demographics. Only GPs and NPs were approached as they deliver the majority of care within NHS primary care; other members of the primary healthcare team such as administrators and receptionists are not normally expected to deal with clinical alerts.
In UK, GPs can be self-employed (GP Partner) or salaried. we approached Partners, Salaried and trainee GPs. NPs, trained specialist nurses, have prescribing rights and are likely to face similar challenges, when providing good quality care, as GPs, yet they may have very different attitudes towards the reminders they experience.
Participants were approached through regional National Institute for Health Research (NIHR) Clinical Research Networks (CRN).[16] A researcher [EC] followed up with participants who showed an interest and invited them to take part in the study. Recruitment, interviews and data coding took place simultaneously. The number of subjects required for the study was dependent on the data. Recruitment ceased when researchers felt no new major themes were immerging. Sixteen clinicians were approached, one GP (NWL) did not respond, fifteen (94%) took part.

Semi-structured Interviews

A single researcher [EC] conducted the face-to-face semi-structured interviews in a setting of the participant's choice. In most cases (13/15) this was at the participant's practice. The topic guide and participant information sheet were developed through literature;[17, 18] meetings between [EC] and

For peer review only - http://bmjopen.bmj.com/site/about/guidelines.xhtml

patients with lived experience; external peer review; and was piloted with a single GP based in North West London (Supplementary file 1). The topic guide was structured to explore three main areas: i) perceptions of the value of the information provided in the reminder; ii) reminder-related behaviours and iii) how to improve reminders. Participant responses were followed-up with probing questions for additional depth and detail. Interviews lasted between 45-60 minutes and was audio-recorded with participant's consent. Participants were paid for their time, which was a set amount determined by CRN guidance. Health Research Authority (HRA) approval was gained (IRAS No: 234951). Ethics approval was not required for this study as participants were NHS staff.

Data analysis

Interviews were anonymised, transcribed verbatim and subjected to a thematic analysis.[19] We went through six stages: 1) familiarisation with data; 2) generating initial codes; 3) searching for themes; 4: reviewing themes; 5) defining and naming themes, and 6) interpretation of themes through consultation with the first, second and third authors (EC, LD, RM). Two researchers (EC and LD) independently coded (stage 2) three transcripts. Eighty percent of codes defined by LD mapped to those defined by EC. Codes that did not map were further examined and recoded if necessary. Codes were then grouped into sub-themes and a thematic map (stage 5) was then developed by researchers [EC, LD, and RM]. We used QSR NVivo 12 to manage data.

Patient and public involvement

We presented the study proposal to a small group of five people, identified as having various health conditions and experience in primary care, as part of an existing advisory group. The patient representatives were reimbursed for their time according to NIHR INVOLVE guidance. The representatives provided feedback on the appropriateness of the research design, research questions, and the topic guide. Their comments were incorporated in study development. For example, the patient representatives felt it was important to ascertain competency in using reminders (how confident they felt in using electronic health records and whether they thought they had received adequate training to manage the reminders) as this is likely to be a barrier to reminder engagement. These questions were added to the topic guide. A single patient representative supported interpretation of the study findings.

For peer review only - http://bmjopen.bmj.com/site/about/guidelines.xhtml

Results.

Participants

Fifteen participants were interviewed; eight were female (53%) and two thirds (67%) were GP partners (Table 2). Eight participants used SystmOne (53%) and seven used Egton Medical Information System (EMIS) clinical administrative system (47%). Participants' years of experience within primary care ranged from less than one to 34 years with a median of 18 years. Participants' practices were on average larger than national practices, median population [interquartile range] 9,355 [4,655 to 14,219] vs 6,681 [4,065 to 10,034). They were, on average, slightly more affluent: median Indices of Multiple Deprivation (IMD) scores were 19 [13 to 28] vs 22 [14 to 32] (where higher scores indicate higher deprivation). Twenty percent of practices (2/10) located within rural populations compared with 15% nationally (1248/8248).

Table 2: Characteristics of interview participants and the administrative systems they use

Attribute	Detail	Number	%
Practicing Region	Y&H	6	40%
	NWL	9	60%
Position	GP Partner	10	67%
	Nurse Practitioner	3	20%
	Salaried GP	1	7%
	GP Trainee	1	7%
Gender	Male	7	47%
	Female	8	53%
Experience (years)	0-1	1	7%
	2-10	2	13%
	11-20	5	33%
	30+	7	47%
Administrative System	EMIS	7	47%
	SystmOne	8	53%

For peer review only - http://bmjopen.bmj.com/site/about/guidelines.xhtml

Study Themes

Participants were familiar with reminders in patients' electronic health records. They reported that reminders were very common and that virtually every patient had at least one occurring during a consultation. The number of reminders varied between patients and were dependent on the patient's health status and time of year.

Our study identified 847 codes within our data which were categorised into sub-themes and four main themes: 1) reaction to a reminder 2) factors influencing the decision to act on reminders (individual and situational), 3) unintended consequences of using reminders, and 4) factors relating to improving reminders (Table 3, Supplementary file 3). Theme examples within the interview data are shown in supplementary file 2.

Reaction to a reminder

Participants' initial reaction to a reminder varied. Although participants found reminders frustrating, there was an overall feeling of acceptance towards reminders. Participant's emotional response did, however, depend on the reminder and the situation.

> *If it's a busy clinic, stress, because it's, like, 'Ugh.' If it looks like it's going to be a complicated patient and it's an out-of-hours reminder, then relief probably.*
> [GP Partner, Y&H, SystmOne]

Use of reminders to organise the patient consultation

Participants reported reminders had become a routine part of their work; they assessed reminders before a consultation and used the information as a guide to organise and prioritise the patient's needs. They used the reminders to build management plans and to relay information to themselves

For peer review only - http://bmjopen.bmj.com/site/about/guidelines.xhtml

or colleagues. Participants talked of feelings of satisfaction, accomplishment and being in control after actioning reminders.

For peer review only

For peer review only - http://bmjopen.bmj.com/site/about/guidelines.xhtml

Table 3: Major themes relating to general practitioner and nurse practitioner views of electronic reminders

1) Reaction to a reminder
**Initial reaction to a reminder**
Dependent on situation
Acceptance
Use of reminders to organise consultation
2) Factors influencing decision to act
**Situational factors influencing decision to act on reminders**
Inappropriate timing of reminder
Time constraints
Oversensitive or invalid reminders
**Individual factors influencing decision to act on reminders**
The perceived value of the advice given
Beliefs reminders promote better care
Experience in professional role
Consultation style
Consequences of ignoring
3) Consequences of using reminders
**Negative consequences of using reminders**
Costs associated with software
Changes clinicians' behaviour
Workload increases
4) Factors relating to improving reminders
**Need to improve number and validity of reminders**
Improving reminder design
Making number of reminders manageable
Standardisation of reminders
**Need to improve efficiency managing reminders**
Training
Team working
Patients' role in managing their conditions

For peer review only - http://bmjopen.bmj.com/site/about/guidelines.xhtml

Individual factors influencing the decision to act on reminder

The perceived value of the advice given

Engagement with the reminders varied. Most participants said they would acknowledge the reminder even if they did not act upon it. The majority commonly stated "70%" were ignored.

> *I wouldn't be able to do anything if I didn't ignore the majority of them.*
> [GP Partner, Male, Y&H, EMIS]

Generally, participants valued the information provided by the reminders.

> *I don't think anybody's ever complained about the serious alerts, it's the infuriating pop-ups*
> [GP Partner, Female, NWL, EMIS]

Some of the participants reported that their views on reminders had changed over time, from feeling overwhelmed to acceptance as they became more familiar with them. Most participants valued the advice given by most reminders.

Some participants felt that prompting for potentially overlooked diagnoses was particularly helpful in the primary care setting. This was due to the high patient turnover and short consultation time, these participants believed recording patient information was automatic while processing the information was sometimes more difficult.

Some participants mentioned current reminders were user-friendly and most believed they were evidence-based which meant they could keep up-to-date with guidance; ultimately improving the care they provided. However, a few participants did not share these views and found all reminders intrusive. Overall, the least popular reminders were the drug cost-saving reminders. Participants complained that the price difference between the previously prescribed drug and its alternative was often minimal.

For peer review only - http://bmjopen.bmj.com/site/about/guidelines.xhtml

Experience and professional role

Participants' feelings towards reminders were often dictated by their professional role, clinical interests, or years of clinical practice. For example, one reported that he provided out-of-hours care to patients outside his normal practice and that the most important reminders were those that conveyed additional patient information because they supported continuity of care. The trainee GP and NPs indicated that drug interaction reminders were particularly useful in supporting safe delivery of care.

Everyone's the same, their interests or their increased fields of knowledge are the ones that they're sort of going to look at more
[Nurse Practitioner, Female, NWL, EMIS]

Many participants accepted that colleagues may have different attitudes towards reminders. For example, they suggested that younger clinicians may find managing reminders easier because they tended to be more computer literate. Yet other participants felt older colleagues would find managing reminders easier as they had more experience to process, act on or ignore the information provided. And nurses were generally more likely to action on reminders because they are less likely to take clinical risk decisions.

… I think doctors are more confident to ignore the prescribing advice than I would be at all.
[Nurse Practitioner, Female, NWL, SystmOne]

There was concern by more clinically experienced participants that reminders undermined their competence. Others felt that GP partners would be more likely to act on the reminders that had financial rewards. However, some felt that by focusing on these reminders it may make patients more sceptical of the care being provided.

I think the partners will really try and crack through the things more. One could argue it's because money is involved.
[GP Partner, Female, NWL, EMIS]

For peer review only - http://bmjopen.bmj.com/site/about/guidelines.xhtml

Participants generally felt the responsibility of actioning a reminder lay with the clinician who was with a patient. However, some seemed to feel more emotionally responsible towards managing the reminders. One participant talked about the guilt associated with failing to action a reminder.

Consultation style

Consultation styles differed across the participants. Some used the computer to support their consultation while others felt that it was important to focus on the patient and only enter data after the patient had left the room. Those who did not use a computer reported that they often missed reminders until it was too late.

> *Writing up oh I should have done that doesn't matter then whether I'm on time or 20 minutes late because they've still gone out the door.*
>
> [GP Partner, Male, Y&H, SystmOne]

Consequences of ignoring a reminder

Participants generally felt they had genuine reasons for ignoring reminders and would document these decisions.

> *I would defend – if I got picked up on it, and I wouldn't often – I would be able to defend why I ignored it for that patient.*
>
> [Nurse Practitioner, Female, NWL, SystmOne]

The consequence of ignoring a reminder differed by reminder type. For example, participants reported that ignoring drug cost-saving reminders resulted in (usually small) financial loses while ignoring drug or allergy reminders could result in a patient safety issue.

> *A lot of them are about money, so ignoring … instruction to use a cheaper drug… you might get a rebuke….Obviously, if it's about patient safety - and a lot of them are about patient safety - but if it feels a true risk and you ignored it then the consequence is that you would do the patient harm.*
>
> [GP Partner, Male, Y&H, EMIS]

Most participants reported there were also opportunistic consequences. For example, a consequence was failing to notify young women of the need for a smear test which could be considered a missed opportunity as these patients rarely visit their GP.

For peer review only - http://bmjopen.bmj.com/site/about/guidelines.xhtml

Some participants' decision to act was influenced by concerns of the consequences of ignoring a reminder. For instance, their behaviour could be highlighted through audit, or ignoring a reminder could lead to a litigation case. NPs felt that they may not get the same support in the case of litigation as GPs and they believed this made them more risk-averse when managing reminders.

> *I'm very risk-averse, and I'm always aware of litigation …. I'm not convinced that the MDU [Medical Defence Union] would defend me in the same way as they would a GP….*
> [Nurse Practitioner, Female, Y&H, SystmOne]

No participants had experienced significant events after ignoring a reminder, but a few participants knew others who had.

Situational factors influencing the decision to act on reminders

Number of reminders

Participants felt that there were too many reminders, which led to irritation or stress. Factors identified to increase the number of reminders were patient age, comorbidities or time of year. Information was also repeated. For example, if a patient had more than one comorbid condition then a reminder to take blood pressure may be listed for each condition

> *No, but you end up just clicking cancel 100 times, it just irritates you in the consultation, because you're typing, kind of examining patients, typing, you've got all this like pop up, pop up, pop up, it's just frustrating*
> [Salaried GP, Female, NWL, SystmOne]

Time constraints

Participants reported they had limited time to manage all reminders in a typical consultation partly due to numbers but also because reminders were time-consuming to action.

Invalid, irrelevant or oversensitive reminders

Participants believed some reminders were irrelevant, did not improve the care they provided or were purely a tick box exercise. Participants also felt irritated that there were errors in some of the information provided.

> *But I know that a lot of those warnings are wrong. They're just simply wrong.*
> [GP Partner, Male, Y&H, EMIS]

For peer review only - http://bmjopen.bmj.com/site/about/guidelines.xhtml

Participants considered some reminders were too sensitive, for example, recording hay fever in the patient's notes could trigger a sepsis alert. In some cases over sensitive reminders were removed.

> *Yeah, it's gone, I think it was flawed. It was coming up on virtually everything so I don't know who decided to switch that off*
> [Nurse Practitioner, Female, Y&H, SystmOne]

Inappropriate timing of reminder

The reason for the patient visit

Participants questioned the appropriateness of the reminders and the need for opportunistic management during the consultation.

> *It's embarrassing, you've got a patient who's coming in who looks like they're in pain or stress and the first thing you have to do is ask about smoking*
> [GP Partner, Female, NWL, EMIS]

Within the consultation

Participants believed the timing of the alert could be made more appropriate to the consultation's natural progression.

> *it would be nice if the reminder came up at the appropriate time, so if you are doing a history … there was a history based reminder, if the medication one came up in the medication, when you press examination maybe all the examination ones so that height, weight, blood pressure came up at that time so that, you know, you could do it in a more natural flowing*
> [GP Partner, Female, NWL, EMIS]

Participants felt that when reminders appeared when the clinician was not with a patient (e.g. drug interaction or drug alternative reminders when managing repeat prescriptions) this was annoying.

In comparison to the last reminder

Participants talked of how the time of year influenced when reminders appeared, for example Quality Outcomes Framework indicators are reset in April. Many felt algorithms could be developed so the reminder's timing was more meaningful and took into consideration when the previous reminder was actioned.

For peer review only - http://bmjopen.bmj.com/site/about/guidelines.xhtml

Changing systems

Participants spoke of frustration in the way systems constantly changed and that not enough precaution was taken to ensure new reminders were fully functional or user friendly.

Negative consequences of using reminders

Reminders are changing behaviour

Participants indicated that reminders have altered GP behaviour within the consultation. It was felt that GP training teaches patient centred care yet some reminders drive behaviour based on financial incentives. Participants felt they and others could become too reliant on reminders. There was also concern that reminders can force an inappropriate action, recording of incorrect information or make them wary of the information they recorded.

> *So, because the CCG [Clinical Commissioning Group] will be looking at our referral letters we should get patient's consent to allow them to look at it, that virtually never happens but you have to tick the box*
>
> [GP Partner, Male, Y&H, SystmOne]
>
> *... they do change the way you do things. So I gave you the example of the sepsis reminders. It makes you wary of putting certain information in because you know it's going to prompt a reminder.*
>
> [GP Partner, Male, Y&H, SystmOne]

Costs and benefits

The participants felt that the reminders suggesting alternatives to cut costs were apparent to the patient and damaged the relationship between patient and clinician. Several participants commented that the cost of the price comparison software probably outweighed any savings.

Increasing workload

Participants pointed out that the alternative drugs prescribed were often unavailable, this required the patient to come back which increased their workload.

For peer review only - http://bmjopen.bmj.com/site/about/guidelines.xhtml

A need to improve the number and perceived value of reminders

Making the number of reminders more manageable

Most participants felt reducing the number of reminders was important. Participants outlined that numbers could be limited by keeping reminders relevant; by removing duplications, which occur when patients have more than one comorbid condition; by removing drug interactions or price comparison alerts for repeat prescriptions and joining up IT systems across the whole of health and social care to prevent duplication of work.

> *Where we're missing information is where things are done outside the practice so, you know, if we had information that was more freely available within the consultation that would prevent duplication, errors, all sorts of things*
> [GP Partner, Female, NWL, EMIS]

Some participants commented that reminders would be less frequent and more relevant if the algorithms used to trigger the reminders were more 'intelligent'.

Standardising and providing more context to a reminder

Participants felt that more context could be given to some reminders. For example, if a patient had previously been advised to have a smear test or a blood pressure measurement is requested because of a high reading at the last visit it would save time going through the notes but also duplication of work. Participants also felt that more use of reminder templates could standardise data recording and help the clinician work more systematically in a consultation.

Improving the design of reminders

Participants repeatedly called for an improvement in the ease of coding to avoid entering text information. Participants suggested the use of hierarchical drop-down lists, for example, with a sepsis alert, lists could speed entering data on the underlying condition.

Most participants did comment that the alert designs made some reminders harder to ignore than others (e.g. red colour coded reminders)

> *They flash and they're big, red angry ones.*
> [GP Partner, Male, Y&H, EMIS]

However, the participants had become accustomed to the format of the reminders so some questioned the importance of a redesign. Colour coding or ranking reminders was felt to be too simplistic to highlight important reminders but helpful to define the type of reminder or where the

For peer review only - http://bmjopen.bmj.com/site/about/guidelines.xhtml

reminder fitted into the progression of a consultation. Participants did feel that redesign of reminders needed to be more individualised to a clinician's consultation style.

I think, ideally, they should be individualised so people consult in different ways, so if I use the computer in a way that I'm not really entering much until the patient has left the room, they don't work for me in that way

[GP Partner, Male, Y&H, SystmOne]

A need to improve the efficiency in managing reminders

Working as a team

The majority of participants felt that reminders were well managed across the practice team, although, some did feel that improvements could be made. For example, they suggested receptionists could be encouraged to offer longer appointments to patients with a high number of quality standard reminders or have dedicated staff to manage them.

It's so difficult to be an acute care clinician plus chronic disease manager at the same time, and that's where alerts sometimes probably get missed or not missed, but consciously avoided. So, an appropriate kind of management of the resource in terms of thinking, right, do we need to have more acute care clinicians or chronic disease managers?

[GP Partner, Male, NW London, EMIS]

Training

All participants commented that new starters, within their practice, received training on using the clinical systems. However, participants felt that the provision of training was variable and many participants felt they needed more training to increase efficiency and to standardise data entry, particularly when new reminders were introduced.

To be honest with you, I think we could all do with training. Like when they update, things like that, I think it would be nice to have a session to say there are recent updates, can all the doctors come and catch up?

[GP Partner, Female, NW London, EMIS]

I don't think it was long enough, the training, because actually EMIS is your… well, computer system is how you do everything

[GP trainee, Female, NW London, EMIS]

For peer review only - http://bmjopen.bmj.com/site/about/guidelines.xhtml

Patient self-management of conditions and data

Participants believed that patients needed to take more responsibility for their own health. They indicated that managing reminders often required signposting patients, and believed that if patients were more aware of their own needs, they could organise their own care. Some participants also believed that patients' access to amend records would have a positive impact on the number of reminders as updating information (e.g. smoking status, QOF reminder) or organising their annual reviews would reduce numbers. Participants also felt that patients would become more aware of drug cost-savings and agree to switch to a cheaper brand if the reminders were available to patients.

For peer review only

Discussion

Key findings

Participants were familiar with the reminders in their clinical systems and felt many were important, providing up-to-date, evidence-based information. Participants' emotional response to the appearance of a reminder depended on the reminder and the situation. Factors influencing the decision to act on the information provided in a reminder related to situational factors (time constraints, appropriateness, reminder sensitivity) and to individual clinician factors (perceived value of advice, GP experience, consultation style). There were negative consequences of using reminders. For example, concerns were reported in becoming over-reliant on the reminders or being forced to record incorrect information. GPs and NPs views on improving reminders were related to firstly a need to improve the efficiency of managing reminders and secondly a need to reduce their number and improve their value.

Comparison with previous research

Our study found similar barriers compared with previous studies.[7-10] These were time pressures in primary care; irrelevant or inappropriate alerts within the clinical workflow; a lack of standardisation of reminders and a need for training to effectively manage the reminders. Clinicians interviewed in England particularly about quality standard reminders,[20] discussed the loss of continuity of care and the increase in workload, but enablers were also reported including more job satisfaction and improved disease management. A study investigating attitudes and motivation of GPs and NPs to deliver public health programmes, found attitudes towards the reminders were mixed. Some participants in the study thought reminders were bothersome, irritating, and many switched them

For peer review only - http://bmjopen.bmj.com/site/about/guidelines.xhtml

off; but others felt they facilitated a consultation and the structured templates ensured essential clinical information was documented.[21] A systematic meta-review (2015),[22] of eleven systematic reviews, suggested four significant challenges that needed to be addressed before clinical decision support systems (which include diagnosis alerts) could fully support the diagnosis. The systems needed to 1) be adaptable and incorporate new knowledge; 2) apply a standardised approach; 3) be deeply integrated with the electronic health record to trigger appropriate timing of the reminder; 4) and have an understanding of the cognitive workflow of the user. Interestingly these findings from using a meta-review methodology closely relate to findings from our qualitative research.

Limitations

In-depth qualitative interviews can offer novel and varied insights into the thoughts and behaviour of participants. We have collected views and experiences from GP and NPs in varying positions based in practices of two regions (North West London, and Yorkshire and Humber) with varying demographic populations. Due to the richness of the data collected, the focused research question, the openness of the participants to discuss their views and the saturation of coding into themes we feel 15 participants was an adequate sample size. We, therefore, feel our findings provide a working hypothesis that is transferable across all GPs and NPs within the English NHS. However, we do acknowledge that parts of the knowledge 'map' may be missing. Primary care clinicians who engaged with the study may have different views than those who did not.

We did not interview practice managers or receptionists who may also be involved in managing reminders within the practice, however, our study focused on obtaining clinician's attitudes to reminders that are specifically delivered at the point of care. We also did not include practice-based pharmacists, who are increasingly being employed in GP surgeries to help to manage long-term conditions or provide advice to patients on multiple medicines. All participants were from practices using either EMIS Web or SystmOne administrative systems. Although, these are the market leaders 10% of practices in England do not use these systems.[23] However, all primary care administrative systems use the same clinical coding system, prescribing classification, appointment system, consultation classification, and core alerts. The systems may slightly differ in the way the reminder is presented and how the clinician interacts with the reminder. For example, some systems may allow the user to 'switch off' the reminder.

We acknowledge that bias could be introduced during the process of data collection, analysis or interpretation. We took steps to limit participant bias by ensuring anonymity and interviewing in a place that was acceptable to the participant. We limited researcher bias by using a peer-reviewed topic guide; having two researchers [LD and RM] to validate coding and co-develop the thematic

For peer review only - http://bmjopen.bmj.com/site/about/guidelines.xhtml

map; involving all authors and a patient representative in interpreting the study findings. Two of the co-authors are practicing GPs [RM and AM] and their interpretations will be informed by practice.

Implications for future development and policy

Studies have discussed the effectiveness of ‘modal’ alerts in prescribing,[24] these are reminders where the system becomes unobtainable behind the alert until actions have been completed, forcing the user to input information. However, our participants found these reminders could be the most irritating, particularly if the information they provided was irrelevant or incorrect. Concerns that incorrect information was often being recorded when an action was forced or that users were altering their behaviour to avoid triggering these reminders, is worrying and needs addressing. However, these types of reminders were valued by more cautious or less experienced clinicians. Modal alerts do have their place, in supporting safe practice, if well designed, using ‘intelligent’ algorithms, and users have appropriate training.

A randomised controlled trial, using computer-simulated patients, found early diagnostic suggestions can improve the accuracy of GPs diagnoses.[25] Our participants commented that processing the information provided by a patient during a consultation could become difficult during busy surgeries and considered reminders to be a useful tool in preventing missed diagnoses.

Our study highlights the need to improve the experience of GP and NPs when interacting with reminders in their patients’ electronic health records. Sophisticated use of data to create the reminders; connecting IT systems across the whole of health and social care; and patients’ access to their data, have all been highlighted as important to progress with the development and improvement of reminders in patient records. The Government’s target to go paperless[26] and introduce a comprehensive system of electronic health records in England by 2020,[27] is a positive move forward in this process. Future development will also need to address different consulting styles used by GP and NPs who will require training to improve the efficiency of managing reminders.

Conclusions

GPs and NPs believe reminders are useful in supporting the provision of good quality care. Improving GPs and NPs’ engagement with reminders centres on further developing their relevance to a GPs

For peer review only - http://bmjopen.bmj.com/site/about/guidelines.xhtml

clinical practice which is personalised, considers cognitive workflow and suppresses inappropriate presentation.

For peer review only

For peer review only - http://bmjopen.bmj.com/site/about/guidelines.xhtml

References

1. Department of Health. *High Quality Care For All: NHS Stage Review Final Report*; 2008.
2. Shojania KG, Jennings A, Mayhew A, Ramsay C, Eccles M, Grimshaw J. Effect of point-of-care computer reminders on physician behaviour: a systematic review. *CMAJ* 2010;**182**:E216-25. https://doi.org/10.1503/cmaj.090578
3. Croskerry P. A universal model of diagnostic reasoning. *Acad Med* 2009;**84**:1022-8. https://doi.org/10.1097/ACM.0b013e3181ace703
4. van der Sijs H, Aarts J, Vulto A, Berg M. Overriding of drug safety alerts in computerized physician order entry. *J Am Med Inform Assoc* 2006;**13**:138-47. https://doi.org/10.1197/jamia.M1809
5. Magnus D, Rodgers S, Avery AJ. GPs' views on computerized drug interaction alerts: questionnaire survey. *J Clin Pharm Ther* 2002;**27**:377-82. https://doi.org/10.1046/j.1365-2710.2002.00434.x
6. Zazove P, Plegue MA, Uhlmann WR, Ruffin MTt. Prompting Primary Care Providers about Increased Patient Risk As a Result of Family History: Does It Work? *J Am Board Fam Med* 2015;**28**:334-42. https://doi.org/10.3122/jabfm.2015.03.140149
7. Zazove P, McKee M, Schleicher L, Green L, Kileny P, Rapai M*, et al.* To act or not to act: responses to electronic health record prompts by family medicine clinicians. *J Am Med Inform Assoc* 2017;**24**:275-80. https://doi.org/10.1093/jamia/ocw178
8. Koskela T, Sandstrom S, Makinen J, Liira H. User perspectives on an electronic decision-support tool performing comprehensive medication reviews - a focus group study with physicians and nurses. *BMC Med Inform Decis Mak* 2016;**16**:6. https://doi.org/10.1186/s12911-016-0245-z
9. Lugtenberg M, Pasveer D, van der Weijden T, Westert GP, Kool RB. Exposure to and experiences with a computerized decision support intervention in primary care: results from a process evaluation. *BMC Fam Pract* 2015;**16**:141. https://doi.org/10.1186/s12875-015-0364-0
10. Medlock S, Eslami S, Askari M, Brouwer HJ, van Weert HC, de Rooij SE*, et al.* Attitudes and experience of Dutch general practitioners regarding computerized clinical decision support. *Stud Health Technol Inform* 2013;**186**:56-60.
11. Kersting C, Weltermann B. Electronic reminders to facilitate longitudinal care: a mixed-methods study in general practices. *BMC Med Inform Decis Mak* 2016;**16**:148. https://doi.org/10.1186/s12911-016-0387-z
12. Dickfos M, King D, Parekh S, Boyle FM, Vandelanotte C. General practitioners' perceptions of and involvement in health behaviour change: can computer-tailored interventions help? *Prim Health Care Res Dev* 2015;**16**:316-21. https://doi.org/10.1017/s1463423614000498
13. European Observatory on Health Systems and Policies. *The Health Systems and Policy Monitor*. URL: https://www.hspm.org/searchandcompare.aspx (accessed 24 November 2019).
14. Bryan C, Boren SA. The use and effectiveness of electronic clinical decision support tools in the ambulatory/primary care setting: a systematic review of the literature. *Inform Prim Care* 2008;**16**:79-91.
15. Jenssen BP, Bryant-Stephens T, Leone FT, Grundmeier RW, Fiks AG. Clinical Decision Support Tool for Parental Tobacco Treatment in Primary Care. *Pediatrics* 2016;**137**. https://doi.org/10.1542/peds.2015-4185
16. National Institute for Health Research. *Local Clinical Research Networks*. URL: https://www.nihr.ac.uk/nihr-in-your-area/local-clinical-research-networks.htm (accessed 18 Jan 2019).
17. Ajzen I. The theory of planned behavior. Organ Behav Hum Decis Process 1991;**50**:179-211. https://doi.org/https://doi.org/10.1016/0749-5978(91)90020-T

For peer review only - http://bmjopen.bmj.com/site/about/guidelines.xhtml

18. Michie S, Richardson M, Johnston M, Abraham C, Francis J, Hardeman W*, et al.* The behavior change technique taxonomy (v1) of 93 hierarchically clustered techniques: building an international consensus for the reporting of behavior change interventions. *Ann Behav Med* 2013;**46**:81-95. https://doi.org/10.1007/s12160-013-9486-6
19. Braun V, Clarke V. Using thematic analysis in psychology. *Qual Res Psychol* 2006;**3**:77-101. https://doi.org/10.1191/1478088706qp063oa
20. Maisey S, Steel N, Marsh R, Gillam S, Fleetcroft R, Howe A. Effects of payment for performance in primary care: Qualitative interview study. *J Health Serv Res Policy* 2008;**13**:133-9. https://doi.org/10.1258/jhsrp.2008.007118
21. Ma RMMN. *What strategies influence general practitioners to deliver public health programmes? An example using the National Chlamydia Screening Programme*: London School of Hygiene and Tropical Medicine; 2017. https://doi.org/https://doi.org/10.17037/PUBS.04645512
22. Nurek M, Kostopoulou O, Delaney BC, Esmail A. Reducing diagnostic errors in primary care. A systematic meta-review of computerized diagnostic decision support systems by the LINNEAUS collaboration on patient safety in primary care. Eur J Gen Pract 2015;**21**:8-13. https://doi.org/10.3109/13814788.2015.1043123
23. Kontopantelis E, Stevens RJ, Helms PJ, Edwards D, Doran T, Ashcroft DM. Spatial distribution of clinical computer systems in primary care in England in 2016 and implications for primary care electronic medical record databases: a cross-sectional population study. *BMJ Open* 2018;**8**:e020738. https://doi.org/10.1136/bmjopen-2017-020738
24. Scott GPT, Shah P, Wyatt JC, Makubate B, Cross FW. Making electronic prescribing alerts more effective: scenario-based experimental study in junior doctors. *J Am Med Inform Assoc* 2011;**18**:789-98. https://doi.org/10.1136/amiajnl-2011-000199
25. Kostopoulou O, Rosen A, Round T, Wright E, Douiri A, Delaney B. Early diagnostic suggestions improve accuracy of GPs: a randomised controlled trial using computer-simulated patients. *Br J Gen Pract* 2015;**65**:e49-54. https://doi.org/10.3399/bjgp15X683161
26. NHS England. *Five Year Forward View*; 2014.
27. Kelsey T, Cavendish W. *Personalised Health and Care 2020: A Framework for Action* National Information Board, Department of Health and Social Care,.

For peer review only

For peer review only - http://bmjopen.bmj.com/site/about/guidelines.xhtml

Version no: 1 19.01.18

Imperial College London

Participant information sheet

Title of Project: Evaluating reminders in primary care electronic patient records

IRAS No: 234951

You are being invited to take part in a research study to evaluate GPs and practice nurses' attitudes towards reminders in electronic patient records. Before you decide it is important for you to understand why the research is being done and what it will involve. Please take time to read the following information carefully and discuss it with others if you wish.
Ask us if there is anything that is not clear or if you would like more information. Take time to decide whether or not you wish to take part.
Thank you for reading this.

Who will conduct the research?

This research is being conducted by Dr Elizabeth Cecil (Senior Researcher) and Professor Paul Aylin (Chief Investigator). The study is part of the Imperial Patient Safety Translational Research Centre's scheme of research. Development of the research has been supported and guided by a group clinicians, patients and researchers with an interest in primary care patient safety.

What is the purpose of the study?

Primary care administrative systems offer opportunity to improve patient care in the form of delivering reminders to primary health care professionals. An electronic reminder is a pop-up prompt created within a clinical administrative system at the point of care. These reminders remind a primary health care professional of an action required to provide good quality care or prevent harm to the patient. Examples of reminders are those that recommend: safe prescribing of medications (and warning of possible interactions); to provide advice on a preventive or clinical intervention (such as smoking cessation or immunisation); or to document clinical information (such as blood pressure). Attitudes to and the impact of reminders have been studied in the US, but little is known about attitudes in the UK. This research will inform further projects within Patient Safety and Translation Research Centre's programme of work to improve administrative systems, which will ultimately contribute to a safer NHS.

Why have I been chosen?

You have been chosen because as a primary health care professional you have extensive experience of using a primary care administrative system and therefore will have important insights into their electronic reminders. For this study we expect to recruit up to 25 further primary care nurses and GPs including partners and salaried GPs who will help us to get a rounded picture of the attitudes towards electronic reminders in primary care.

Do I have to take part?

No. It is up to you to decide whether or not to take part. If you do decide to take part you will be given this information sheet to keep and be asked to sign a consent form. If you decide to take part you are still free to withdraw at any time and without giving a reason.

What will happen to me if I take part?

If you decide to take part you will be asked a series of open ended questions to assess your views on reminders, which will be recorded for analysis. These questions will asked in a single interview, which can take place in a setting of your choice and will take a maximum of 1hr of your time (including the brief, consent and debrief). You will be reimbursed for your time and travel.

For peer review only

Version no: 1 19.01.18

What if something goes wrong?

If you are harmed by taking part in this research project, there are no special compensation arrangements. If you are harmed due to someone's negligence, then you may have grounds for a legal action. Regardless of this, if you wish to complain, or have any concerns about any aspect of the way you have been treated during the course of this study then you should immediately inform the senior researcher (e.cecil@imperial.ac.uk). The normal National Health Service complaints mechanisms are also available to you. If you are still not satisfied with the response, you may contact the Imperial AHSC Joint Research Compliance Office.

What are we studying

We are investigating attitudes towards reminders in primary care administrative systems. A reminder is a pop-up prompt created within a clinical administrative system at the point of care. These reminders remind a primary health care professional of an action required to provide good quality care or prevent harm to the patient. Examples of reminders are those that recommend: safe prescribing of medications (and warning of possible interactions); to provide advice on a preventive or clinical intervention (such as smoking cessation or immunisation); or to document clinical information (such as blood pressure).

What are the possible disadvantages of taking part?

We know that as a practising GP/Nurse your time is a limited. We will do our utmost not to inconvenience you and will ask for only 45 minutes of your time.

What are the possible benefits of taking part?

We cannot promise the study will directly affect your relationship with reminders in primary care, but the information could help to improve clinical systems which will ultimately contribute to a safer NHS.

Will my taking part in this study be kept confidential?

All information collected in both the consent form and the interview data will be kept strictly confidential. You will be assigned a code and only the senior researcher will have the key to identify participants. Personal data contained within the consent form will only be available to direct members of the study team and will be stored within the restricted secured networked IT system at Imperial College for 10 years after the completion of the study. The audio recordings of the interviews will be transcribed by a transcription company, Page Six, who have a confidentiality agreement in place with Imperial College London. The original recordings will be destroyed after transcription. Our procedures for handling, processing, storage and destruction of their data are compliant with the Data Protection Act 1998.

What will happen to the results of the research study?

Interviews will be anonymised using thematic analysis. Results will be written up into reports and research articles. These will include anonymised quotes from interviews. You will be notified of these before publication.

Who is organising and funding the research?

This study is part of the work of the Patient Safety and Translational Research Centre at Imperial College London (PSTRC). PSTRC is funded by the National Institute for Health Research.

Who has reviewed the study?

This study was reviewed by members of the PSTRC Research Partnership Group which comprises of patients and members of the general public, on 19.12.17. The Study Coordination Centre has obtained approval from the Health Regulator Authority (HRA).

Who should I contact for further information?

The senior researcher, Elizabeth Cecil can be contacted at:

e.cecil@imperial.ac.uk
020 7594 1838

Imperial College London,
Department of Primary Care and Public Health, Reynolds Building, St Dunstan's Road
London W6 8RP

For peer review only

Version no: 1 19.01.18

Imperial College London

Topic Guide

Title of Project: Evaluating reminders in primary care electronic patient records

IRAS No: 234951

Design: Semi-structured interview

Objectives:

- To assess GPs and practice nurses' familiarity with reminders in electronic health records.
- To determine GPs and practice nurses' views on the relevance of information provided by reminders in electronic health records.
- To understand the factors involved in why GPs and practice nurses ignore some reminders and not others.
- To explore how GPs and practice nurses feel reminders can be improved.

Brief:

Thank you very much for taking part in this study. My name is Elizabeth Cecil I work at Imperial College London. As you know we are carrying out a project to assess the primary health care professionals' views on reminders in electronic health records in primary care.

An electronic reminder is a pop-up prompt created within a clinical administrative system at the point of care. These reminders remind a primary health care professional of an action required to provide good quality care or prevent harm to the patient. Examples of reminders are those that recommend: safe prescribing of medications (and warning of possible interactions); to provide advice on a preventive or clinical intervention (such as smoking cessation or immunisation); or to document clinical information (such as blood pressure).

The interview is very informal and confidential. Only me and the direct members of my team will see or hear it. Your name will not appear on anything that we write and you will be notified before we publish the results. With your permission I would like to record the interview. This so that I can concentrate on your responses rather be distracted by taking notes. All recordings will be destroyed once the project is complete. Right, we'll both sign the two consent forms and there is a copy for you and one for me. Thank you we'll start. *Start taping.*

For peer review only

Version no: 1 19.01.18

Questions	Possible probes
**I would like to start by collecting some information on you and the practice**	
Please confirm your participant ID	It's written on your consent form
What is your position at this practice?	Are you a salaried GP, GP partner or practice nurse?
How long have you been qualified?	How long as a GP/practice nurse
What EHR system is used at this practice?	
Do you feel confident using the EHR system in your clinical practice?	Compared to your colleagues how competent do you feel you are?
**I now wish to discuss your familiarity with electronic reminders (knowledge)**	
For this research we define a reminder (in electronic primary care patient records) as: **A prompt created within a clinical administrative system at the point of care which reminds a primary health care professional of an required or desired action.**	
Can you give me some examples of reminders that you come across in your working day?	Are these the most common reminders
How many reminders do you tend to come across in a single day?	What factors are related to the number of reminders? E.g. time of year, patient age, comorbidities
Do reminders come up at other times than at the point of care, for example when the GP or administrator are adding patient information into the EHR?	Who is ultimately responsible for the outcome of a reminder if it does not always appear at point of care?
**(Skills)**<br>Do you feel confident to effectively manage reminders?	Is the training the GPs receive in the surgery or in their training elsewhere?
Have you been given training on how to respond to reminders?	
**Now I wish to explore your attitudes to reminders (beliefs – self-standards)**	
What do you think about reminders?	
How credible or relevant do you find the information provided by reminders systems?	
Can you give me an example of a reminder that you feel is particularly useful/relevant?	Why are they useful?<br>How much do you rely on these prompts?
**(To explore behaviour towards reminders / intentions)**	(Emotional response)

For peer review only

What is your immediate reaction to a reminder when it appears on your screen?	How does this make you feel e.g (delight, stress, dread)
Are there some reminders you are more likely to override/ignore than other others?	Why do you override/ignore this reminder compared with other reminders?
Can you expand on what factors dictate you to override/ignore?	For example are you more likely to ignore at the end of a day/week
Are there some reminders you never override/ignore?	Can you expand on why?
How often do you override/ignore reminders?	How many would that be, say, out of 10?
**(Beliefs about consequences)**<br>What will happen if you ignore a reminder?	
Can you give any example of a negative outcome after ignoring an alert?<br>How was this handled and was anything learned after the event?	If not by you but by any of your colleagues?
**(Social influences)**<br>To what extent does practice management or your peers influence your response to reminders?	
**(Decision processes)**<br>Do you feel that reminders benefit the care you provide in the consultation?	Do the reminders help you with the decisions you make on treating the patient?
Do you think there is convincing evidence to support the use of reminders in electronic health records?	
You have given me a detailed description of your attitudes towards reminders.	
Do you think your colleagues both within your practice and within the profession feel the same way?	What dictates why they feel differently about reminders?
**(Constraints)**<br>What additional resources do you feel could help you effectively respond to a reminder?	Time is an obvious constraint but are there others?
**Lets discuss your opinions on how to improve reminders**	
How would you improve the reminders if you could?	What specific changes do you feel would help facilitate the use of electronic reminders?
Do you feel that a redesign should include a ranking system?	For example colour coding for importance.
What information do you feel needs to be included that may have previously been missed?	

For peer review only

Version no: 1 19.01.18

Do you think these improvements will change your behaviour to and attitudes towards reminders?	Will you be less likely to ignore a reminder?

The Patient Safety and Translational Research Centre is carrying out research identify determinants of deterioration in primary care patients using EHRs.

Do you think that there are already reminders that can help to identify deteriorating patients or those patients where a diagnoses has previously been missed?	If not a reminder what other tools do you use?
Do you think that a reminder to flag up a deteriorating patient would be important?	

Debrief

Thank you for taking part. Do you have any questions that you want to ask me? If you do need to get hold of me in the future, my contact details are on the information sheet.

For peer review only

Themes and subthemes	Examples	Participant
**Reaction to a reminder**		
Acceptance	*I think that at this stage it doesn't make me feel anything because it's there all the time. I think it's become fairly automatic that you glance at it and it depends which one of the many warnings it is.*	[GP Partner, Male, Yorkshire & Humber, EMIS]
Dependent on situation	*If it's a busy clinic, stress, because it's, like, 'Ugh.' If it looks like it's going to be a complicated patient and it's an out-of-hours reminder, then relief probably.*	[GP Partner, Male, Yorkshire & Humber, SystmOne]
Use of reminders to organise consultation	*What I tend to do, and I think most doctors do this, is we kind of do a pre-consultation before the list. So then, I would go through my list, see what reminders come up, and think, okay, fine, supposing it's a child, I'll remember to talk to Mum about reminding about immunisations.*	[GP Partner, Female, NW London, EMIS]
**Individual factors influencing decision to act on reminders**		
The perceived value of the advice given		
Up to date advice	*So, the guidance is very accurate and up-to-date and regularly, it's responsive with regards to the changes that are happening in the BNF to be updated*	[GP Partner, Male, NW London, EMIS]
With infrequent preventive care	*the reminders for cervical smears, because it's three years so some people just don't receive the letters; so promoting them …*	[GP Registrar, Male, NW London, EMIS]
Supportive	*And I think doctors are more confident to ignore the prescribing advice than I would be at all*	
The value of reminders to organise work		*I wouldn't … do half the things that I do if it wasn't for the reminders.*
Beliefs that reminders promote better care		
For continuity of care	*I appreciate them, I think that they have definitely a use because I can't remember everything*	[GP Partner, Female, NW London, EMIS]
For possible diagnoses	*if there's a kind of brain fatigue of seeing so many patients, it's easy sometimes to record, but not necessarily process, what you are writing. That's in any field. So, you're writing, yes, heart rate 150, I know that's high but I haven't really thought about it. So, if the sepsis alert comes up, I think it's good because it also has made you think.*	[GP Partner, Male, NW London, EMIS]
Experience in professional role, eg. Clinical interests or years in clinician practice		
Beliefs of one's personal capabilities	*I mean, I'd be worried if a GP at this stage wasn't aware of sepsis*	[Salaried GP, Female, NW London, SystmOne]

For peer review only

Beliefs of others confidence capabilities		[Nurse Practitioner, Female, NW London, SystmOne]
Feelings of being in control	*I will try, like this morning, I've got quite a few I've done the whole lot of, and I feel really satisfied.*	[Nurse Practitioner, Female, NW London]
Interests and belief in importance of reminder	*everyone's the same, their interests or their increased fields of knowledge are the ones that they're sort of going to look at more, so even if they are improved, things like the patients with rheumatoid arthritis and things that need reviews*	[Nurse Practitioner, Female, NW London, EMIS]
Position and importance of reminder	*I think the partners will really try and crack through the things more. One could argue it's because money is involved*	[GP Partner, Female, NW London, EMIS]
Feelings of responsibility	*Doctors …they might ignore the foot check whereas myself or my colleagues, peers, would either do it or tell somebody to go and book an appointment*	[Nurse Practitioner, Female, NW London, SystmOne]
Feelings of guilt	*I will feel guilty, if I haven't asked somebody whether they smoke I feel a twinge of guilt, but clinically it's probably not going to do a great deal*	[Nurse Practitioner, Female, Yorkshire & Humber]
Consultation style		
	Writing up oh I should have done that doesn't matter then whether I'm on time or 20 minutes late because they've still gone out the door.	[GP Partner, Male, Yorkshire & Humber, SystmOne]
Consequences of ignoring reminders		
Litagation	*.... people are very reluctant to make a change because... nobody wants to take responsibility. Everybody's scared of being sued and the computer companies are very scared of being sued... so they overdo it. They absolutely overdo it. And unfortunately that therefore makes the warnings much weaker because they're not specific enough.*	[GP Partner, Male, Yorkshire & Humber, SystmOne]
Litagation	*I'm very risk-averse, and I'm always aware of litigation.*	[Nurse Practitioner & Admin Role, Female, Yorkshire & Humber, SystmOne]
Patient Harm	*if it's about patient safety - and a lot of them are about patient safety - but if it feels a true risk and you ignored it then the consequence is that you would do the patient harm.*	[GP Partner, Male, Yorkshire & Humber]
	… and the patient's not in front of you so to change a medication when they're not there… albeit it's the same drug, it's clinically not… I don't think it's safe. So, yes, I override them.	[GP Registrar, Female, NW London, EMIS
Financial loss	*A lot of them are about money, so ignoring the CCG's instruction to use a cheaper drug, sometimes you might get a rebuke if it's a really, really expensive decision. But that would be as rare as hen's teeth that that would happen.*	[GP Partner, Male, Yorkshire & Humber, EMIS]
Lost opportunity	*example cervical screening, and I can't remember a case off the top of my head. But if you*	[GP Partner, Male, NW London, EMIS]

For peer review only

	ignored it and then you didn't necessarily remind the patient and you think, this is a young person who doesn't really come into the surgery, this was an opportunistic consultation, it would have been a great chance to remind them of that,	
Increase workload	*Well ultimately, you have to call the patient back in to get the work done, so ultimately yes.*	[Nurse Practitioner, Female, NW London, SystmOne]
Audit	*I mean I was told that they do audit who's doing the flus so who's choosing to say, no, come back but then they can look at that and see was it appropriate because obviously if they're unwell*	[Nurse Practitioner, Female, NW London]
Genuine reasons for ignoring reminders	*I would defend – if I got picked up on it, and I wouldn't often – I would be able to defend why I ignored it for that patient.*	[Nurse Practitioner, Female, NW London, SystmOne]
**Situational factors influencing decision to act on reminders**		
Time constraints	*I wouldn't be able to do anything if I didn't ignore the majority of them.*	[GP Partner, Male, Yorkshire & Humber, EMIS]
	That's pretty standard, I mean here they do 10 minutes and every hour you get a 10 minute block so they say that's 12 minutes.	[Nurse Practitioner, Female, NW London, EMIS]
Inappropriateness of reminder's appearance		
Within consultation flow	*I think they're fantastic if they're at the right time.*	[GP Partner, Male, Yorkshire & Humber, SystmOne]
Within consultation flow	*And instead of them just being, for example, a box somewhere it would be nice if the reminder came up at the appropriate time, so if you are doing a history if the appropriate reminder came up in… there was a history based reminder, if the medication one came up in the medication, when you press examination maybe all the examination ones so that height, weight, blood pressure came up at that time so that, you know, you could do it in a more natural flowing*	[GP Partner, Female, NW London, EMIS]
Within consultation flow	*Some of the other prompts tend to come afterwards rather than at the point of care,*	[GP Partner, Male, Yorkshire & Humber, SystmOne]
In relation to reason for patient visit	*It's embarrassing, you've got a patient who's coming in who looks like they're in pain or stress and the first thing you have to do is ask about smoking -*	[GP Partner, Female, NW London, EMIS]
In relation to the time of year	*I'm not going to respond to anything to do with the flu jab at this point in time because it's the wrong time of year.*	[GP Partner, Female, NW London, SystmOne]
	with QOF related indicators there is a timeframe and, … there may be a reminder to do something that was only done two or three months ago, for which there is no real clinical need to do now but because the system is set up in that way..	[GP Partner, Male, NW London, EMIS]

In timing since previous reminder	*And so do you think that a reminder should have a sort of additional algorithm that says basically six months afterwards*	[GP Partner, Male, NW London, EMIS]
Oversensitive or invalid reminders	*Extremely sensitive. So, if you put in something like hay fever, it will come up with a sepsis alert, because it will just catch that bit that you say fever, so a lot of time you end up just going exit, exit, exit and it actually creates more work. I don't think my consultations have ever benefitted from a sepsis alert, never.*	[GP Registrar, Female, NW London, EMIS]
System changes	*So, for example, atrial fibrillation has a new template that's come out on the EMIS and I just had a patient today with atrial fibrillation and I noticed it wasn't working properly. So then, I would say to – I rang the manager to say, "Can we just go through this at one point?" Because I think if I'm having problems with it, we probably are as a group because it doesn't seem to be uploaded properly.*	[GP Partner, Female, NW London, EMIS]
**Negative consequences of using reminders**		
Costs associated with software outway any benefits	*The original people that did the alerts were appalling and there was evidence that the cost of the alerts was way in excess of the savings …. So there's lots of examples in the NHS where we make really poor financial decisions. Popups can be one of those.*	[GP Partner, Male, Yorkshire & Humber, EMIS]
Permanency of data recorded	*And I know other patients, even a doctor colleague who had something put on her child's records because she was widowed and so the child was considered more vulnerable. And fair enough, so somebody else looking out for them and thinking about their needs, but it was put on in such a way that it looked like the child was in need on a protection plan, you know, being mishandled. And she couldn't get it taken off for love nor money even though she was a perfectly competent parent and there was a perfectly reasonable need in that child being more vulnerable because her child's father had just died. So there are pop-ups that can be damaging, actually.*	[GP Partner, Female, NW London, EMIS]
Changes PHCPs behaviour		
Changes in the way data is recorded	*they do change the way you do things. So I gave you the example of the sepsis reminders. It makes you wary of putting certain information in because you know it's going to prompt a reminder.*	[GP Partner, Male, Yorkshire & Humber, SystmOne]
Distracts PHCP from patient centredness	*they've just got to be careful that they're not too much of a burden and too intrusive all the time, because it then does detract you away from your primary objective, which is, kind of, interacting with that patient,*	[GP Partner, Male, Yorkshire & Humber, SystmOne]
Patients aware reminders impact on behaviour		
Damages patient-PHCP relationship	*But a lot of patients also have an awareness that it earns us money. And they will quote that back at you. So, you have to be careful and you have to, you know… I think…*	[Nurse Practitioner & Admin Role, Female, Yorkshire & Humber, SystmOne]

Workload increases as patients consult more	*and it's exactly the same medication just a different brand, so then you're spending more time explaining to them, "It's exactly the same thing". So, as I said, I appreciate why it's there; sometimes I do switch from the generic brand to the branded because it's whatever is in fashion and it's common, but there are*	[GP Partner, Male, NW London, EMIS]
Learning from mistakes	*It's not about blame. How can we avoid this? And we put strategies*	[Nurse Practitioner, Female, Yorkshire & Humber, SystmOne]
**Need to improve number and validity of reminders**		
Making number of reminders more manageable		
Joining up IT systems across health and social care	*where we're missing information is where things are done outside the practice so, you know, if we had information that was more freely available within the consultation that would prevent duplication, errors, all sorts of things*	[GP Partner, Female, NW London, EMIS]
Intelligent algorithms	*It's a bit annoying because something like that a lot of the alerts will come up if there's ever been a problem so you look back and you think oh I might actually ask the patient saying about your, you know, liver function and they'll go, 'Oh that was four years ago and it's all fine now and I used to drink and I don't anymore' but EMIS… I don't know how far back EMIS goes but it seems to pick up anything abnormal but you have to check it.*	[Nurse Practitioner, Female, NW London, EMIS]
Patient self-management of condition	*You would hope a lot of them, like blood tests when you're on medication that need to check would like to think that the patient would take the onus themselves and know I've got to do this every three months or I've got to do this every six months but sadly a lot of them…*	[Nurse Practitioner, Female, NW London, EMIS]
Patient self-management of data	*I mean, I think some of the QOF ones, the minor things like smoking and everything, if we go to sort of patient-held IT records and things like this, they will actively put that stuff in for us*	[Nurse Practitioner, Female, Yorkshire & Humber, SystmOne]
Make all reminders valid	*I don't think anybody's ever complained about the serious alerts, it's the infuriating pop ups.*	[GP Partner, Female, NW London, EMIS]
	So, I think if you say proceed, then it doesn't ask anything, but if you say ignore, it comes up saying, basically, why are you ignoring me? So, that can be quite frustrating, especially if you're thinking, you should realise why I'm ignoring this after a while	[GP Partner, Female, NW London, EMIS]
Make reminders more timely	*with QOF related indicators there is a timeframe and, as I said, there may be a reminder to do something that was only done two or three months ago, for which there is no real clinical need to do now but because the system is set up in that way..*	[GP Partner, Male, NW London, EMIS]
Standardisation of reminders	*I'd like an NHS formulary that was set nationally*	[GP Partner, Female, NW London, EMIS]

For peer review only

**Need to improve the validity of reminders**		
Improving the evidence base of reminders	*QOF, the evidence-base for that is poor, you know, not robust. And I expect that the evidence-base for this will be mixed. But I don't think it's going to mean that they go away.*	[Nurse Practitioner, Female, Yorkshire & Humber, SystmOne]
Reminders force incorrect data entry	*So, because the* CCG [Clinical Commissioning Group] *will be looking at our referral letters we should get patient's consent to allow them to look at it, that virtually never happens but you have to tick the box*	[GP Partner, Male, Yorkshire & Humber, SystmOne
Need to improve efficiency managing reminders		
Training	*… no, sometimes extra training to know what's the quickest way of doing something, …., so if it's recording, say, a vaccination that you've given, it might say that they need a B12 is it quicker to use templates?*	[GP Partner, Female, NW London, EMIS]
	…. I don't think it was long enough, the training, because actually EMIS is your… well, computer system is how you do everything; it's your note system, it's how you communicate with other healthcare professionals, it's your referral system, and you learn to use it very superficially	[GP Registrar, Female, NW London, EMIS]
**Need to improve the design of reminders**		
Use of drop down	*and then you could actually have something saying it's an ENT, it's a chest, it's an acute abdomen, it's a urine, you know, and then you could just quickly pick which one you want and then you could put that information in because the advantages of that is that those would be just a tick box exercise whereas when you add it yourself you would put in urine analysis then you have a drop down then you have to find it, you know? So, you could just free text it but that's ridiculous, you don't want to free text anything, you want to read code as much as you possibly can.*	[GP Partner, Female, NW London, EMIS]
Better use of icons	*They could be personalised, not quite as funkily as this where it's very obvious to me within a flash that this patient smokes - she uses an inhaler and she's probably got restrictive or obstructive or interstitial lung disease - that she's got brittle bones, well she's definitely got lung disease, that she's at risk for dementia because (overspeaking), that she might not have had her flu jab. I mean, I can tell very, very quickly. I never need to ask somebody whether they smoke or not; I just look at the screen...So the icons are quite useful in that respect.*	[GP Partner, Female, NW London, SystmOne]
Need to change attitudes	*I think the alerts are just going to be annoying going forward until we as clinicians change the way that we have to input data*	[GP Registrar, Female, NW London, EMIS]
Ranking in reminders isn't very easy	*Ranking on what basis? It's hard to know whether a patient's allergy is more important than a child's protection need or a patient's, you know, medication review*	[GP Partner, Female, NW London, SystmOne]

For peer review only

Reorganisation of chronic disease care	*At the moment, there's a real kind of shift in what a lot of GPs are doing at the moment, so it's moved from, historically, a lot of chronic disease management and now it's a lot of acute care. It's getting increasingly difficult to be both kinds of doctor in the ten minutes. So, to respond to reminders, in theory, for the best interests of patients, you've got to be thinking, can chronic disease management be managed in a different way with stable patients, it's like almost a chronic disease birthday or something like that where the patient thinks, right, every year in the month of my birthday, the practice is going to deal with all my reminders to do with diabetes or whatever chronic disease it is. It's so difficult to be an acute care clinician plus chronic disease manager at the same time, and that's where alerts sometimes probably get missed or not missed, but consciously avoided. So, an appropriate kind of management of the resource in terms of thinking, right, do we need to have more acute care clinicians or chronic disease managers?*	[GP Partner, Male, NW London, EMIS]

For peer review only

Supplementary Figure 1: Thematic map of general practitioner and nurse practitioner views of electronic reminders

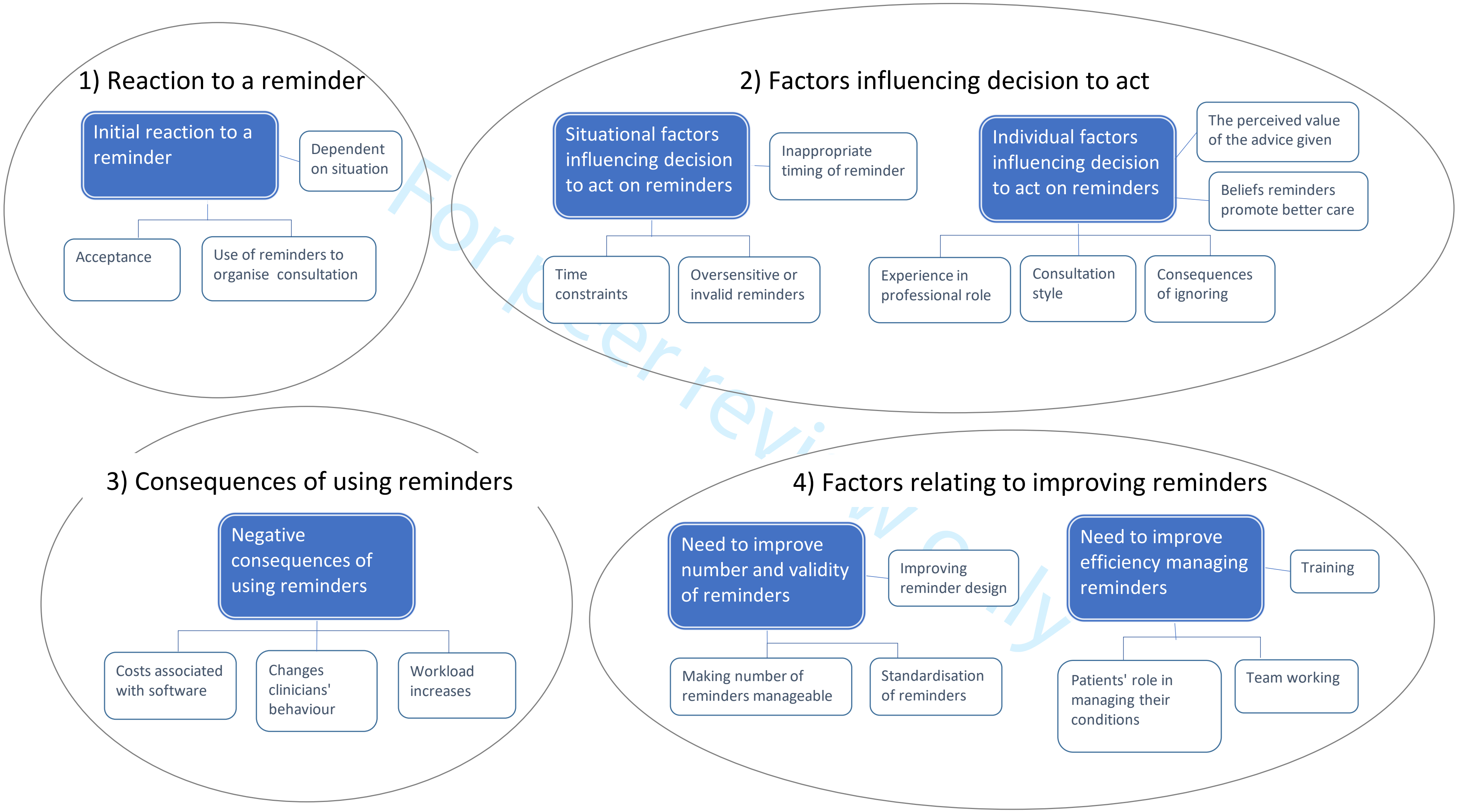

COREQ (COnsolidated criteria for REporting Qualitative research) Checklist

A checklist of items that should be included in reports of qualitative research. You must report the page number in your manuscript where you consider each of the items listed in this checklist. If you have not included this information, either revise your manuscript accordingly before submitting or note N/A.

Topic	Item No.	Guide Questions/Description	Reported on Page No.
**Domain 1: Research team and reflexivity**			
Personal characteristics			
Interviewer/facilitator	1	Which author/s conducted the interview or focus group?	
Credentials	2	What were the researcher's credentials? E.g. PhD, MD	
Occupation	3	What was their occupation at the time of the study?	
Gender	4	Was the researcher male or female?	
Experience and training	5	What experience or training did the researcher have?	
Relationship with participants			
Relationship established	6	Was a relationship established prior to study commencement?	
Participant knowledge of the interviewer	7	What did the participants know about the researcher? e.g. personal goals, reasons for doing the research	
Interviewer characteristics	8	What characteristics were reported about the inter viewer/facilitator? e.g. Bias, assumptions, reasons and interests in the research topic	
**Domain 2: Study design**			
Theoretical framework			
Methodological orientation and Theory	9	What methodological orientation was stated to underpin the study? e.g. grounded theory, discourse analysis, ethnography, phenomenology, content analysis	
Participant selection			
Sampling	10	How were participants selected? e.g. purposive, convenience, consecutive, snowball	
Method of approach	11	How were participants approached? e.g. face-to-face, telephone, mail, email	
Sample size	12	How many participants were in the study?	
Non-participation	13	How many people refused to participate or dropped out? Reasons?	
Setting			
Setting of data collection	14	Where was the data collected? e.g. home, clinic, workplace	
Presence of non-participants	15	Was anyone else present besides the participants and researchers?	
Description of sample	16	What are the important characteristics of the sample? e.g. demographic data, date	
Data collection			
Interview guide	17	Were questions, prompts, guides provided by the authors? Was it pilot tested?	
Repeat interviews	18	Were repeat inter views carried out? If yes, how many?	
Audio/visual recording	19	Did the research use audio or visual recording to collect the data?	
Field notes	20	Were field notes made during and/or after the inter view or focus group?	
Duration	21	What was the duration of the inter views or focus group?	
Data saturation	22	Was data saturation discussed?	
Transcripts returned	23	Were transcripts returned to participants for comment and/or	

For peer review only

For peer review only - http://bmjopen.bmj.com/site/about/guidelines.xhtml

Topic	Item No.	Guide Questions/Description	Reported on Page No.
		correction?	
**Domain 3: analysis and findings**			
Data analysis			
Number of data coders	24	How many data coders coded the data?	
Description of the coding tree	25	Did authors provide a description of the coding tree?	
Derivation of themes	26	Were themes identified in advance or derived from the data?	
Software	27	What software, if applicable, was used to manage the data?	
Participant checking	28	Did participants provide feedback on the findings?	
Reporting			
Quotations presented	29	Were participant quotations presented to illustrate the themes/findings? Was each quotation identified? e.g. participant number	
Data and findings consistent	30	Was there consistency between the data presented and the findings?	
Clarity of major themes	31	Were major themes clearly presented in the findings?	
Clarity of minor themes	32	Is there a description of diverse cases or discussion of minor themes?	

Developed from: Tong A, Sainsbury P, Craig J. Consolidated criteria for reporting qualitative research (COREQ): a 32-item checklist for interviews and focus groups. *International Journal for Quality in Health Care*. 2007. Volume 19, Number 6: pp. 349 – 357

Once you have completed this checklist, please save a copy and upload it as part of your submission. DO NOT include this checklist as part of the main manuscript document. It must be uploaded as a separate file.

For peer review only
